# Comprehensive nutritional analysis of 95 oat cultivars reveals large variability in nutritional profile: protein, starch, fat, β-glucan and fibre

**DOI:** 10.1038/s41538-026-00800-z

**Published:** 2026-03-17

**Authors:** Nefeli Lampoglou, Atikur Rahman, Ewen Mullins, Aylin W. Sahin, Cathal McCabe, Elke Arendt, André Brodkorb, Daniela Freitas

**Affiliations:** 1 Teagasc Moorepark Food Research Centre, Fermoy, Co. Cork, Ireland; 2 School of Food and Nutritional Sciences, University College Cork, Cork, Ireland; 3 Teagasc, Crop Science Department, Carlow, Ireland; 4 School of Agriculture and Food Science, University College Dublin, Dublin, Ireland; 5 APC Microbiome Ireland, University College Cork, Cork, Ireland

**Keywords:** Biochemistry, Plant sciences

## Abstract

Nutritional composition determines whether oats can deliver established health benefits like cholesterol reduction, which requires specific β-glucan thresholds unachievable by consuming a standard serving of typical commercial cultivars. Current yield-focused breeding overlooks the substantial nutritional diversity within the species, limiting consumer access to nutritional profiles optimised for different nutritional targets and leaving the potential for enhanced health outcomes untapped. This study characterised the nutritional composition of 95 Irish-grown oat cultivars and compared field (2024) versus glasshouse (2023) grown samples from 21 cultivars. Key components showed wide variation, with fat displaying the largest range and starch the most consistency, with concentrations ranging as follows: protein 11.5–22.9%, starch 47.2–65.3%, fat 2.4–10.0%, ash 1.4–2.9%, β-glucan 2.8–6.6%, and calculated fibre-rich fraction 8.5–26.9% (dry matter basis). Glasshouse-grown samples had significantly higher protein, fibre-rich fraction, and ash than field-grown counterparts (*p* < 0.001). These findings reveal untapped potential for breeding oats with targeted nutritional profiles to support dietary recommendations and food industry applications.

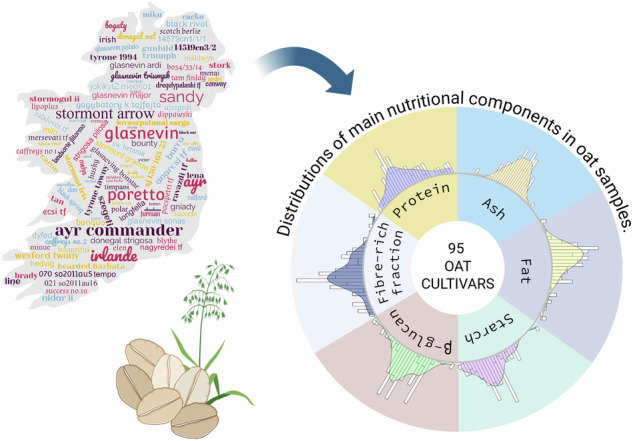

## Introduction

Oat (*Avena sativa*) is a versatile, promising crop that has recently gained attention for human consumption thanks to its proven health benefits. The European Food Safety Authority (EFSA) has approved three health claims linking oat fibre to: (1) reduction of blood cholesterol^1^; (2) reduction of post-prandial glycaemic responses^2^; (3) increase in fecal bulk^3^. These health benefits have been confirmed through extensive research, as previously reviewed^4–8^. At the same time, the research community has been exploring further potential benefits, such as prebiotic effects^7,9,10^, antioxidant activity^7,8^, phenolic-mediated gut microbiota modulation^11^, maintenance of a healthy body weight^4,6,7^ and improved immune functions^6,7,12^.

Rising consumer awareness of the health benefits related to oat consumption is driving the increase in demand that has been observed in recent years. Specifically, annual oat consumption reached 0.86 kg per capita globally in 2021, representing an almost 40% increase from 2016 (https://www.fao.org/faostat/). Accordingly, worldwide efforts for higher yield have proven fruitful, as the global average yield has more than doubled from 1.3 tonnes per harvested hectare in 1961 to 2.8 tonnes per hectare in 2022 (https://www.fao.org/faostat/). The annual world production of oats is now around 22 million tonnes. Of these, 34% (an estimated 7.6 million tonnes) was produced in the European Union in 2024, making it the largest oat producer globally (https://www.fas.usda.gov/). Ireland alone produced more than 227,000 tonnes of oats (https://teagasccropreport.ie/reports/harvest-report-2024). The main varieties grown in Ireland are Husky, Isabel and, until recently, Barra^13^.

Despite the growing recognition of oat health benefits driving consumer demand, nutritional quality is not integrated into grain quality assessments for production, cultivation, or agricultural decisions. While some nutritional parameters, such as the protein content, are considered for certain grains, such as wheat, grain quality assessments for oat are centred on agricultural performance metrics. These typically include thousand-grain weight, kernel content, and hectolitre weight. In particular, the Irish Department of Agriculture, Food and the Marine (DAFM) uses these three factors as grain quality determinants to rate and recommend oat varieties for sowing each year^13^. Other agronomic parameters taken into consideration include yield, disease resistance, lodging and earliness of ripening^14^ as well as factors related to grain appearance, damage and presence of foreign materials^15^. Previous research on 120 genotypes has shown that substantial genetic variation was observed for agronomic traits such as lodging, yield, and thousand grain weight^14^. Population structure analyses of global oat collections further reveal moderate genetic diversity and geographic clustering among landraces and cultivars, highlighting untapped variation for trait improvement^16^. Large-scale compositional surveys have further established baseline variability in oats, with a recent 2025 study of elite North Dakota breeding lines documenting ranges for nutritional traits including protein, beta-glucan, oil, and fibre, as well as milling qualities^17^. While field performance is of paramount importance when growing crops, neglecting the nutritional composition of the various cultivars can have far-reaching consequences. This oversight can result in the underutilisation of varieties richer in health-promoting components such as β-glucan, consequently limiting consumers’ access to oat with an optimal nutritional profile. Additionally, it may constrain farmers’ ability to tap into emerging high-value markets that prioritise specific nutritional traits to meet evolving, health-conscious consumers’ preferences and personalised nutrition needs. This approach risks eroding the available genetic pool. Long-term breeding of a small number of consistently performing well-adapted cultivars can lead to a narrowing of genetic diversity in many crops. For oat specifically, this genetic narrowing can have direct implications for the health benefits that consumers can obtain. A clear example can be observed in the Irish market, where oat-only products typically contain around 2.4–4.3% of β-glucan and 11–13.7% of protein, with carbohydrates accounting for up to 62–74% of the total composition. According to EFSA, 3 g of oat β-glucan should be consumed daily for blood cholesterol reduction^1^. Moreover, 4 g of oat (or barley) β-glucans for each 30 g of available carbohydrate need to be consumed per meal to reduce the post-prandial glycaemic response^2^. In practice, these targets are challenging to meet with typical consumption patterns and the common options available on the market. Considering that a standard portion of oats is 40 g, typically containing around 1 g of β-glucan, one would need to consume the equivalent of three standard portions (120 g) daily to reach the required β-glucan amount for cholesterol regulation. Consequently, despite the increases in production and consumption, most consumers are unlikely to obtain an adequate intake of oat-derived nutrients to fully benefit from their health-promoting effects.

However, the literature indicates that it is unrealistic to assign single fixed values to oat composition, as each component can vary widely within a broad range^18–21^. For example, β-glucan content as high as 8% has been reported^22^. Differences in the molecular weight and structure of β-glucan may have an effect on the viscosity of the digestive chime, resulting in differences in the measured glycaemic index and blood cholesterol^5,23–25^. Furthermore, variation in the molecular structure of starches from different varieties resulted in varied starch digestion rate, which can have implications on the glycaemic index and blood glucose rise^26^. Moreover, varieties bred specifically for high protein content can reach up to 24% protein content on dry weight^27^. Apart from the variation of nutrient composition from cultivar to cultivar, differences in the quality and structure of some components have also been observed. In the case of protein, as a result of breeding for high total protein content, the major increase happens in the globulin fraction that is richer in essential amino acids such as lysine—the limiting amino acid in cereals^27^. Hence, higher-protein varieties may also have a more balanced amino acid profile. Recent reviews confirm that cereal proteins, including those from oats, serve as valuable nutritious alternatives offering nutraceutical benefits that enhance nutritional quality and functionality for sustainable health applications^28,29^.

Although research on both agronomic and nutritional quality continues advancing, integration between these fields remains limited. Consequently, breeding programmes that target both agronomic performance and nutritional quality remain underexplored. There is a clear need to bring these areas together, as traits can be genetically correlated, meaning that selecting for a particular agronomic trait may inadvertently affect a nutritional trait, and vice versa. One challenge, for example, is the relationship between β-glucan and yield, which have been shown to be negatively correlated^23,30^. However, this relationship is not absolute. Cervantes-Martinez et al.^30^ observed that even though there was a tendency for the high β-glucan varieties to have a lower yield, that was not the case for all of them; some lines were high in β-glucan while also having comparable agronomical characteristics with the controls^30^. This indicates that breeding both for high β-glucan and high yield is possible, but further research and more integrated breeding approaches are needed to fully realise this potential.

The vast pool of genetic resources created through generations of breeding worldwide and the existing natural variation of the oat grains give us access to heterogeneous collections of cultivars. Expanding grain quality considerations to include nutritional composition can be expected to increase the nutritional diversity of the crop. This may have important repercussions from a public health perspective, as it can result in providing consumers access to a diverse range of products with varying amounts, combinations, and ratios of health-promoting components. Therefore, this study aimed to (1) investigate the underexplored variation in nutritional composition among oat cultivars by characterising key components among 95 cultivars and (2) examine whether this variation is consistent within field and glasshouse growing conditions. Identifying genotypes with superior nutritional profiles and establishing a knowledge database can inform future breeding efforts for favourable nutritional quality, depending on the application and target composition.

## Results

This section presents the compositional analysis of oat cultivars grown under field and glasshouse conditions. First, the variation in key nutritional components among field-grown cultivars is described, followed by correlations among nutritional components. Variations in growing conditions among field-grown sample groups (F21, F22 and F24) impose inherent limitations on cross-year comparisons. Nevertheless, providing a global overview of the entire sample set remains valuable for deriving meaningful insights into compositional patterns, given the large and diverse set of cultivars analysed. Therefore, results are presented with both within- and cross-group analyses used as appropriate. Finally, the compositional profiles of glasshouse-grown samples are presented and compared to their field-grown counterparts (Fig. 4). All results are reported on a dry matter basis unless otherwise specified.

### Compositional analysis of field-grown samples

**Fig. 1:**
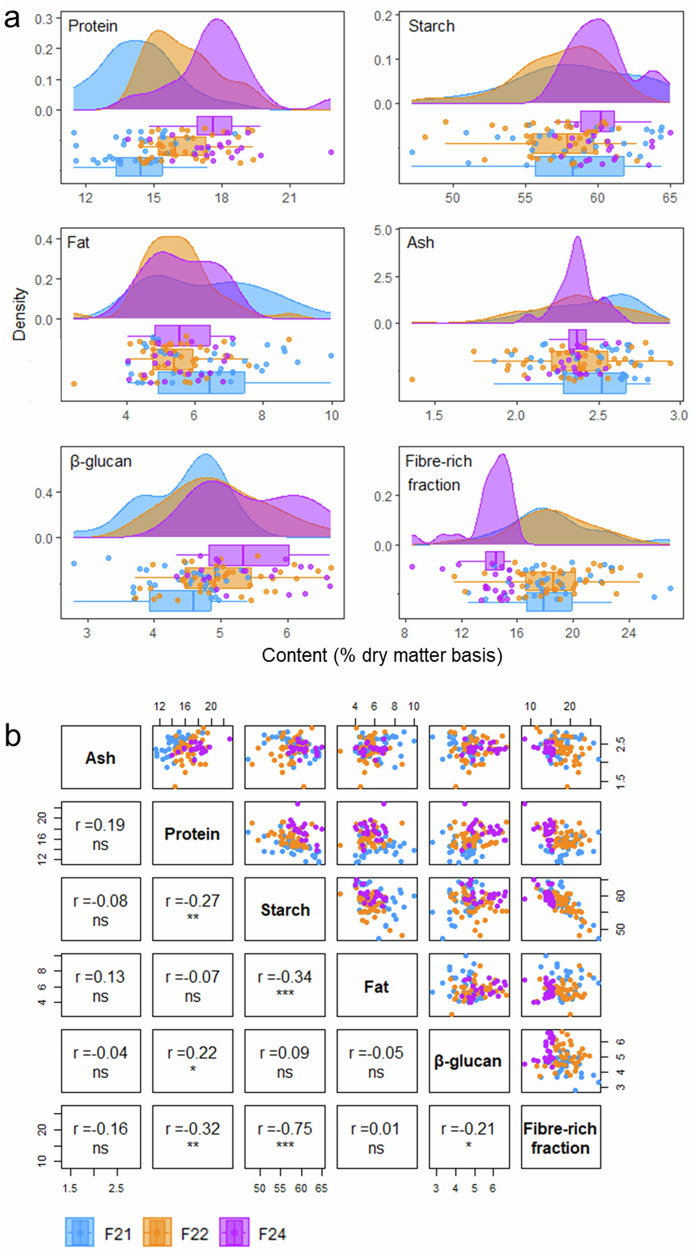
Distributions and correlations of nutritional components across 95 oat cultivars. **a** Variations in protein, starch, fat, ash, β-glucan and calculated fibre-rich residue. The half-violin plots (filled curves) represent the data distribution. Boxplots display median (thick vertical line), interquartile range (box), and whiskers (to 1.5× interquartile range or data extremes). **b** Correlation plots showing Pearson correlation coefficients, statistical significance (*, **, ***, ns for *p* < 0.05, <0.01, <0.001, and ≥0.05, respectively) and scatterplots. Scatterplots (circular markers) in both panels represent individual cultivar means (% dry matter basis). Colour: blue—Field 2021 (F21); orange—Field 2022 (F22); purple—Field 2024 (F24). The residual “fibre-rich fraction” was calculated by difference, subtracting the sum of protein, fat, total starch, ash, and moisture from the total sample weight (100%).

**Fig. 2:**
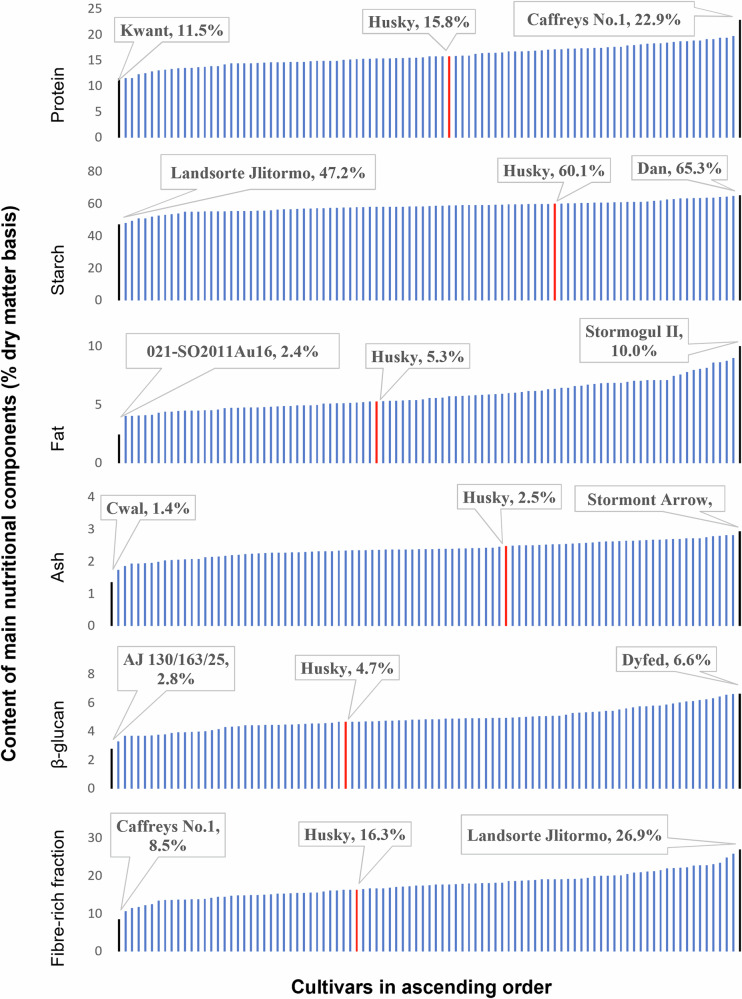
Macronutrient composition of 95 oat cultivars. Cultivars are arranged in ascending order per nutrient (expressed as percentage on a dry matter basis). The highest and lowest performing varieties, along with the reference cultivar (Husky, red bars), are highlighted in the callouts. Error bars have been removed for clarity; the complete version, including analytical variation, is presented in Fig. S1. The residual “fibre-rich fraction” was calculated by difference, subtracting the sum of protein, fat, total starch, ash, and moisture from the total sample weight (100%).

**Fig. 3:**
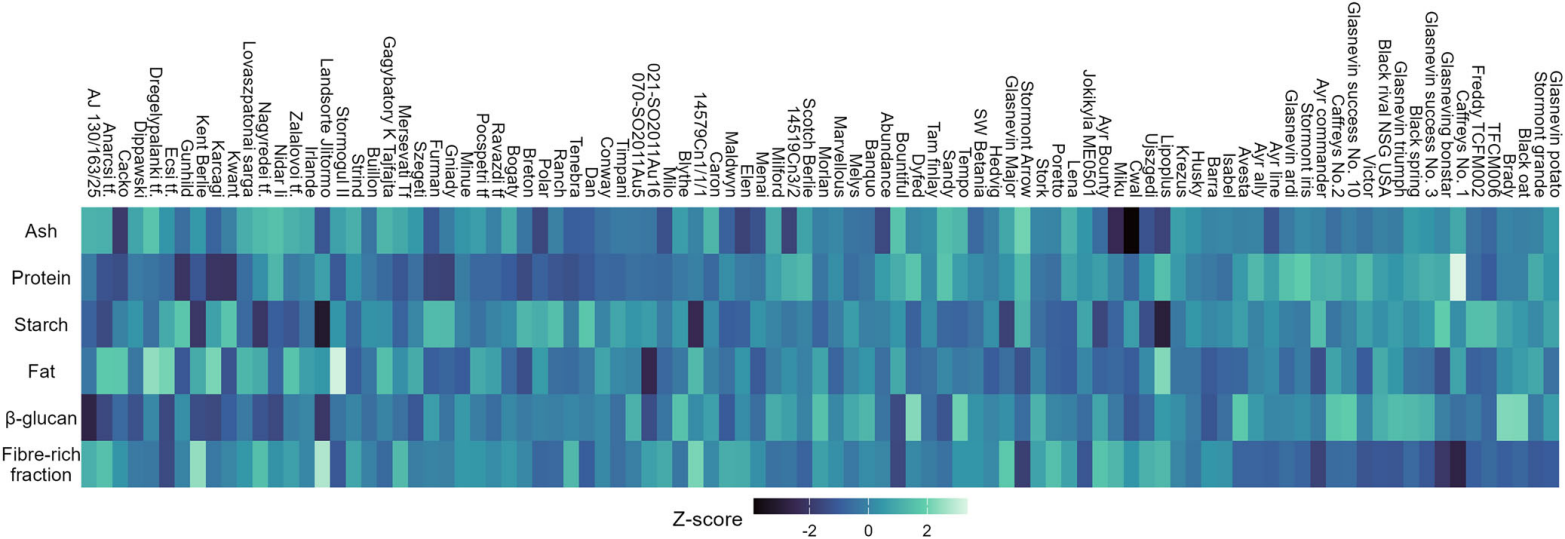
Heatmap of nutritional composition variation across 95 field-grown oat cultivars from sample groups F21, F22, and F24. Cell colours represent *z*-scores calculated separately for each nutritional trait across all cultivars, with the colour scale shown in the legend. Darker colours indicate lower values. The residual “fibre-rich fraction” was calculated by difference, subtracting the sum of protein, fat, total starch, ash, and moisture from the total sample weight (100%).

The compositional variation among the 95 field-grown oat cultivars analysed is illustrated in Fig. 1a, which displays separate raincloud plots for protein, starch, fat, ash, β-glucan, and calculated fibre-rich fraction. Each plot provides a comprehensive visualisation of the data distribution (half-violin plots in the upper area of each graph), central tendency (box plots), and individual data points (mean values for each cultivar depicted as scatter plots) within each group of field-grown cultivars. These results are summarised in Table 1. Figure 2 shows ordered bar charts of compositional data for each cultivar, illustrating the range across all field-grown samples irrespective of harvest year. The cultivars at both extremes, along with the reference variety Husky, are annotated. Error bars (standard deviation) are omitted for clarity. The complete version of the figure is provided as Fig. S1 in the Supplementary Material. Full compositional data and homogeneous groups are provided in Table S2. Figure 3 is a heatmap highlighting the nutritional composition variation across all field-grown samples. It acts as a tool to visualise cultivar-level patterns of the dataset. The magnitude and range of variation in compositional traits across all three field-grown groups, representing three different harvest years and distinct sets of cultivars, were substantial and broadly comparable (Table 1). This is also evident in Fig. 1a, where the distributions of most analysed nutritional components show a high degree of overlap. The coefficients of variation of each component followed similar trends between the years (Table 1), with fat being consistently the most variable component while starch was the least variable. The consistency of this diversity, regardless of changes in genetic pool or growing year-system, underscores the inherent variability across the analysed oat cultivars. Because both the cultivar composition and environmental conditions differed between years, formal statistical comparisons of trait values across years have not been conducted, as any observed differences may reflect both genetic and year-to-year influences.

**Table 1:** Contents of protein (*N* $\times$ 5.83), starch, fat, ash, β-glucan, and fibre-rich fraction (calculated by difference) as percentage on dry matter basis, per field-grown sample group as well as all field samples together

	F21 (Field 2021)			F22 (Field 2022)			F24 (Field 2024)			All field samples		
Number of samples	33			41			21			95		
	**Average** ± **SD (%)**	**Range (%)**	**CV (%)**	**Average** ± **SD (%)**	**Range (%)**	**CV (%)**	**Average** ± **SD (%)**	**Range (%)**	**CV (%)**	**Average** ± **SD (%)**	**Range (%)**	**CV (%)**
Protein	14.34 ± 1.66	11.48–18.59	11.6	16.31 ± 1.48	14.40–19.38	9.1	17.68 ± 1.85	13.87–22.85	10.5	15.93 ± 2.06	11.48–22.85	12.9
Starch	58.33 ± 4.30	47.17–65.29	7.4	57.34 ± 3.14	48.07–62.66	5.5	60.33 ± 2.18	57.01–64.98	3.6	58.35 ± 3.58	47.17–65.29	6.1
Fat	6.32 ± 1.62	4.03–10.00	25.7	5.46 ± 1.06	2.45–8.75	19.3	5.61 ± 0.96	4.03–7.11	17.2	5.79 ± 1.31	2.45–10.00	22.6
Ash	2.45 ± 0.26	1.86–2.82	10.5	2.34 ± 0.31	1.36–2.94	13.3	2.37 ± 0.12	2.07–2.63	5.2	2.39 ± 0.26	1.36–2.94	11.1
β-glucan	4.43 ± 0.60	2.80–5.40	13.6	4.97 ± 0.72	3.72–6.65	14.5	5.42 ± 0.73	4.32–6.62	13.4	4.88 ± 0.77	2.80–6.65	15.8
Fibre-rich fraction	18.56 ± 3.24	12.49–26.93	17.5	18.54 ± 2.90	11.46–24.80	15.6	14.01 ± 1.77	8.48–15.59	12.6	17.54 ± 3.38	8.48–26.93	19.2

Data presented as the average of three replicates ± SD and coefficients of variation (CV).

The moisture content was determined to enable the expression of all other components on a dry matter basis, ensuring consistency and comparability by accounting for water variability. The moisture content of the field-grown samples ranged from 8.5% to 12.8%, with a mean of 10.6 ± 0.9 and a CV of 8.4%. The distribution of moisture content was normal in all examined sample groups (*p* = 0.732, *p* = 0.645, *p* = 0.077 for F21, F22, F24, respectively).

Protein, estimated from the nitrogen content using a nitrogen conversion factor of 5.83, ranged from 11.5 ± 0.2% (Kwant, F21) to 22.9 ± 0.1% (Caffreys No.1, F24), as seen in Fig. 2. The average protein across all field groups was 15.9 ± 2.1%. Protein content followed a normal distribution for F21 and F24 groups but not for F22, where it appears positively skewed (Fig. 1a). The CV for protein content across all samples was 12.9%. Husky, which served as the reference cultivar, had an average protein content (15.8 ± 0.1%), which was lower than that of 46% of the cultivars analysed.

The starch content ranged from 47.2 ± 4.4% (Landsorte Jlitormo, F21) to 65.3 ± 3.6% (Dan, F21), showing the lowest variability among all analysed components (Fig. 2, CV = 6.1%). The distribution of starch content was normal for groups F21 and F24, but not F22, because of two outlying low values that make the distribution appear negatively skewed (Fig. 1a). Husky had a starch content of 60.0 ± 3.0%, which was higher than that of 69% of the samples.

Fat content ranged between 2.4 ± 0.4% (021-SO2011Au16, F22) and 10.0 ± 0.1% (Stormogul II, F21), with an overall mean of 5.8 ± 1.3% (Table 1). The reference variety had an average fat content (5.3 ± 0.0%, Fig. 2). The distribution of fat content was normal but appeared positively skewed in F22 (Fig. 1a), with the majority of samples clustered toward the lower end of the range. Fat showed the greatest variability among all measured components across the entire sample set (CV = 22.6%), a pattern that was also observed within each group.

Ash content ranged from 1.4 ± 0.6% (Cwal, F22) to 2.9 ± 0.0% (Stormont Arrow, F22), as seen in Fig. 2, with an average of 2.4 ± 0.3% and a CV of 11.1%. The distribution of ash content did not follow a normal distribution for any of the sample groups (*p* = 0.054, *p* = 0.235, *p* = 0.544 for F21, F22, F24, respectively, Fig. 1a). Ash content of the variety Husky was average (2.5 ± 0.1%).

β-glucan content ranged from 2.8 ± 0.3% (AJ 130/163/25, F21) to 6.6 ± 0.6% (Dyfed, F22) with an overall mean of 4.9 ± 0.8%. The CV was 15.8%. Husky, the reference variety, had an average β-glucan content equal to 4.7 ± 0.3% (Fig. 2), which was lower than that of 62.1% of the cultivars analysed. The distribution of β-glucan content was normal for field samples harvested in groups F22 and F24, but non-normal (*p* = 0.046) and bimodal in F21 (Fig. 1a). Nevertheless, the mean β-glucan contents were similar across all three field-grown sample groups, with overlapping ranges and comparable coefficients of variation, indicating comparable levels of variation within each group despite differences in data distribution.

The fibre-rich fraction varied between 8.5 ± 2.5% (Caffreys No.1, F24) and 26.9 ± 4.6% (Landsorte Jlitormo, F21), with a mean value of 17.5 ± 3.4%. Fibre-rich fraction content followed a normal distribution in groups F21 and F22 but not in F24 (*p* < 0.001) (Fig. 1a). The reference variety, Husky, had a value of 16.3 ± 4.1%, lower than that of 61% of the field-grown cultivars included in this study (Fig. 2).

### Variation among cultivars

The variation of the nutritional composition was assessed separately within each group by comparing field-grown samples from the same harvest year (F21, F22 or F24). Differences in nutrient profiles among cultivars were observed across all groups (MANOVA, *p* < 0.001, *η*^2^_*p* = 0.903 (F21), 0.864 (F22), 0.845 (F24)), indicating variation among cultivars. Following the significant MANOVA, univariate ANOVAs on technical triplicates revealed marked differences among cultivars (*p* ≤ 0.001) for all nutrients within each group (Table S5). Post hoc analysis (Tukey’s test) identified multiple distinct groupings at *α* = 5%, shown as superscript letters in Table S2. These analytical groupings reflect clear compositional separation within each sample group, emphasising cultivar-level variability under identical growing conditions. The number of distinct groups varied between nutrients and sample groups: protein and fat showed the greatest subdivision, forming 14 to 19 groups and 11 to 20 groups, respectively, while β-glucan formed 2 to 8 groups, starch 4 to 5 groups and the calculated fibre-rich fraction 3 to 5 groups. This variation aligns with the observed coefficients of variation (CV) across nutrients, with fat and protein exhibiting higher CVs, and thus greater subdivision into significant groups, while starch and fibre-rich fraction showed fewer groups, reflecting limited variability. This compositional variation across cultivars is clearly visualised in Fig. 3, where the heatmap reveals distinct nutritional patterns among oat genotypes, allowing for diverse combinations of nutrient profiles.

### Compositional analysis of glasshouse-grown samples

The compositional profiles of the 21 cultivars grown under glasshouse conditions (G23) are summarised in Table 2 and presented for each individual cultivar in Table S3. The distributions of all components of the group G23 were normal, as confirmed by the Shapiro–Wilk test (protein *p* = 0.327, total starch *p* = 0.279, fat *p* = 0.082, ash *p* = 0.663, β-glucan *p* = 0.202, calculated fibre-rich fraction *p* = 0.856). The average protein content was 20.1 ± 1.8%, ranging from 17.0% to 22.9%. The average contents of starch, fat and ash were 52.3 ± 4.3%, 4.5 ± 1% and 2.9 ± 0.2%, respectively. The fibre-rich fraction ranged from 13.9% to 28.2% with a high variation (CV = 18.1%), while β-glucan varied from 3.4 to 5.2% with a medium variation (CV = 13.1%). The lowest coefficients of variation were observed for ash and starch, at 8.0% and 8.3% respectively, and the highest for fat (CV = 22.4%). Fibre-rich fraction and β-glucan presented intermediate CV levels of 18.1% and 13.1%, respectively. Significant overall differences in compositional profiles were observed among cultivars (MANOVA, *p* ≤ 0.001), followed by univariate ANOVAs on technical triplicates that revealed between-cultivar differences (*p* < 0.001) for all nutrients except the ash content. Post hoc analysis (Tukey’s test) identified multiple homogeneous groups at *α* = 5%, presented as superscript letters in Table S3. Notably, β-glucan content and fibre-rich fraction showed greater variability in glasshouse-grown samples, with 4 and 6 homogeneous groups identified by the post-hoc test, compared to 2 and 3 in field samples. On the contrary, ash, fat, starch, and protein varied more in the field-grown cultivars (3, 11, 5 and 14 groups, compared to 1, 9, 2 and 12, respectively).

**Table 2:** Contents of protein (*N* $\times$ 5.83), starch, fat, starch, ash, β-glucan, and fibre-rich fraction (calculated by difference) as percentage on dry matter basis of glasshouse-grown oat cultivars

	G23 (Glasshouse 2023)		
Number of samples	21		
	**Average** ± **SD (%)**	**Range (%)**	**CV (%)**
Protein	20.10 ± 1.80	17.01–22.85	9.0
Starch	52.29 ± 4.32	41.02–59.65	8.3
Fat	4.54 ± 1.01	3.21–7.00	22.4
Ash	2.88 ± 0.23	2.43–3.44	8.0
β-glucan	4.21 ± 0.55	3.41–5.23	13.1
Fibre-rich fraction	20.61 ± 3.73	13.90–28.21	18.1

Data presented as average of three replicates ± SD, and coefficients of variation (CV).

**Fig. 4:**
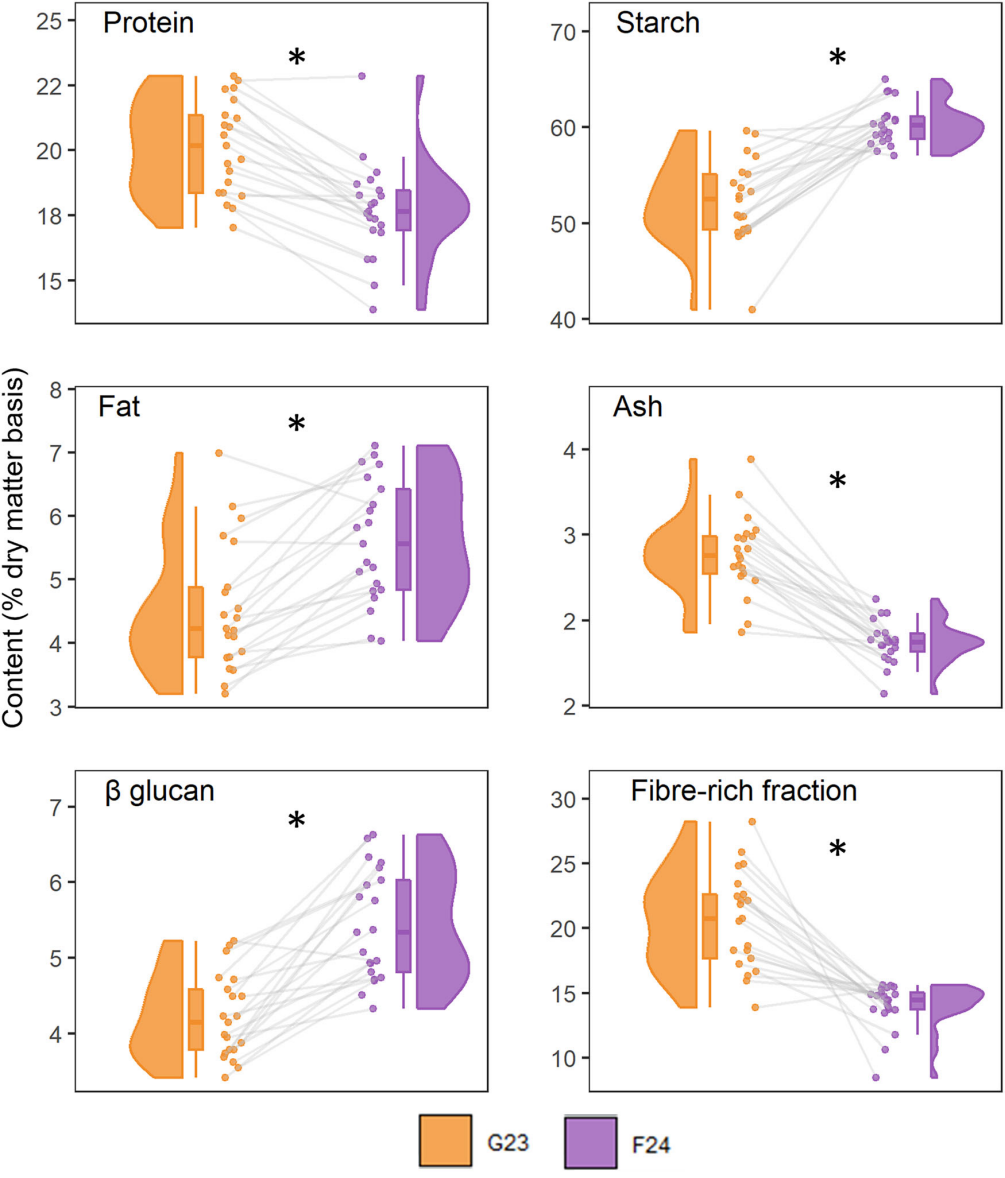
Comparison of the distributions of nutritional components across 21 glasshouse- (G23) vs. field-grown (F24) cultivars. Linked raincloud plots show protein, starch, fat ash, β-glucan and calculated fibre-rich residue. Half-violin plots (filled curves) represent the data distribution; boxplots display median (thick horizontal line), interquartile range (box), and whiskers (to 1.5× interquartile range or data extremes); scatterplots (circular markers) show individual cultivar means (% dry matter basis). Asterisks indicate statistically significant differences (*p* < 0.001, paired *t*-test) between sample groups. The residual “fibre-rich fraction” was calculated by difference, subtracting the sum of protein, fat, total starch, ash, and moisture from the total sample weight (100%).

Figure 4 presents a comparative view of the distributions and data points of the cultivars grown in the glasshouse (G23) and in the field (F24). A paired *t*-test showed that all components were significantly different between the two sample groups (*p* < 0.001). Specifically, ash, protein, and fibre-rich fraction contents were statistically significantly higher in the glasshouse-grown samples, while fat, starch, and β-glucan were higher when samples were grown on the field. The connected raincloud plots also allow visual assessment of cultivar ranking across sample groups. Where connecting lines are broadly parallel, cultivar rankings are largely conserved between years. In contrast, extensive crossing and tangling of lines indicate substantial changes in ranking. Cultivar ranking was largely stable for protein but varied for the β-glucan.

### Correlations among nutritional components

Correlation coefficients were initially calculated separately for each field group to enable comparisons among varieties grown under the same conditions (ST4a-c). Significant correlations were observed for the following: starch and fat in F22 and F24 (*r* = −0.45, *p* = 0.003 and −0.64, *p* = 0.002); starch and protein in all groups (F21: *r* = −0.62, *p* < 0.001; F22: *r* = −0.33, *p* = 0.037; F24: *r* = −0.51, *p* = 0.017). Correlations among the other components were less consistent.

To examine which relationships were consistent across the entire dataset, correlation coefficients were also calculated for the full field-grown sample set (95 cultivars), revealing weaker but significant correlations, specifically starch and fat (*r* = −0.34, *p* < 0.001) and starch and protein (*r* = −0.27, *p* = 0.008). These relationships are visualised in Fig. 1b through scatterplots for each nutrient pair, providing an overview of trait-trait relationships. The correlation coefficients are also provided in Table S4d.

The negative relationship between starch and protein was also observed in the glasshouse-grown samples (*r* = −0.55, *p* = 0.010, Table S4e). None of the other significant relationships found in F24 was present in G23.

Some correlations were observed between the calculated fibre-rich fraction and other components, including starch in groups F21 (*r* = −0.81, *p* < 0.0001 8·10^−9^), F22 (*r* = −0.77, *p* < 0.0001) and GH24 (*r* = −0.86, *p* < 0.0001); ash in groups F22 and F24 (*r* = −0.43, *p* = 0.005 and *r* = −0.48, *p* = 0.028, respectively). However, these are largely driven by the arithmetic structure of the difference calculation under the closed-sum constraint, rather than independent biological variation. Consequently, these correlations should not be over-interpreted as evidence of a direct biological trade-off between starch and fibre-related fractions.

## Discussion

This study provides a comprehensive analysis of the compositional diversity among a broad range of oat cultivars grown in Ireland. A total of 95 oat cultivars were grown in the field, with 21 of these also grown in a glasshouse. Significant variation among cultivars was observed within all groups for key nutritional components, including protein, fat, starch, β-glucan, and calculated fibre-rich fraction (based on technical triplicates). Differences due to year-growth system combinations were also evident; for the subset of varieties grown both in field and glasshouse, all nutritional components differed between these year-management-growth systems. This consistent diversity across varying cultivar sets and year-management-growth conditions demonstrates substantial nutritional profile variation among oat cultivars.

The field-grown oat cultivars included in this study were separated into three groups (F21, F22 and F24) based on harvest year (2021, 2022 or 2024, respectively). Despite each group consisting of a distinct set of cultivars, the magnitude and range of compositional variation were similar across years, as shown by the extent of overlapping distributions in Fig. 1a. As the results indicate, substantial variation was observed across all cultivars within each field-grown sample group. In each group, both differences in the overall nutrient profiles (*p* < 0.001) and for each component (*p* ≤ 0.001) were confirmed by MANOVA and individual ANOVAs on technical triplicates, respectively. Cultivar differences in the nutritional composition of oat cultivars were therefore analytically pronounced, which corroborates the initial hypothesis of substantial inter-varietal variation.

The compositional profiles of the 21 cultivars grown under field (Table 1, group F24) and glasshouse conditions (Table 2) are shown as connected raincloud plots in Fig. 4. All components differed significantly between the two conditions (*p* < 0.001, paired *t*-test). Ash, protein, and calculated fibre-rich fraction were higher in glasshouse-grown samples, whereas fat, starch, and β-glucan were higher in field-grown samples. Moreover, the variation of individual components differed between the two growth systems; for example, the fibre-rich fraction varied more in glasshouse-grown samples, whereas starch content varied more in field samples. This indicates that cultivar performance under glasshouse conditions (growth system, year, management) does not fully reflect field management systems. Still, the observed trends provide useful insight into the direction of change for specific compositional traits and can be useful for studies of the potential of glasshouse screening for nutritional qualities. The cultivar ranking across the two conditions was also assessed. The stability of protein ranking suggests strong genetic control and lower sensitivity to variation arising from differences in growth system, management, and year. In contrast, the marked changes in β-glucan ranking indicate greater environmental responsiveness, highlighting the influence of combined year–system effects on this trait. To our knowledge, while there are comprehensive studies on oats grown under either field or glasshouse conditions, no study has directly compared the nutritional composition of the same cultivars across both systems. Previous research using glasshouse-grown oats has typically focused on specific traits rather than overall composition, and results have often revealed discrepancies when compared to field outcomes. For example, breeding for high fat content in glasshouse-grown oats was not effective^31^. Similarly, copper fertilisation significantly increased yields under glasshouse conditions, but had no effect in the field^32^. Additionally, drought trials in glasshouse settings showed inconsistent results compared to field results, and were therefore considered unreliable^33^. These examples highlight the limitations of glasshouse trials in replicating field performance, underscoring the need for direct comparisons of nutritional composition across systems. These comparisons offer a straightforward approach to assessing the range of variability that can be expected under different growth systems, which helps to better capture the overall potential of each cultivar.

Previous studies have also observed the effect of cultivar and growth conditions on the composition of oats for key components. In earlier research, Welsh et al.^34^, after growing 60 oat varieties in four locations over three consecutive years, noted that even though the protein content would change among the growing locations and years, the relative content of the varieties did not change significantly^34^. Similar studies, using smaller sample sizes (8–18 cultivars), observed that both factors affected the protein content significantly^18,21,35–37^. Nevertheless, in practice, the environmental effect does not seem to substantially impact the results. Doehlert et al.^36^ found that the protein content of 12 cultivars varied from 14.6 to 19.6% (genotypic means) across 12 environments, while the respective environmental means were 15.3-19%. For other components, the literature presents conflicting results. For instance, starch content has been reported to be influenced more by the environment^36^, the genotype^18^, or neither^37^. Similarly, it is unclear whether the fat content is influenced more by the environment^18,38^, the genotype^21,35,36^, or both^37^. β-glucan appears to be influenced by both factors^18,36,37^, though some studies report only a genotypic effect^21,35^.

Direct comparisons of results across different studies are challenging, as the observed environmental effects on oat composition depend on the specific range and nature of the conditions in each study, as well as the susceptibility of the examined oat cultivars to these effects. The wide diversity in the nutritional components analysed in our study is evident regardless of growth system, management, year, or genetic background. The substantial ranges observed and the overlap across sample groups therefore confirm the potential for selection and improvement in a variety of conditions and genetic pools, and reinforce the value of screening for nutritional quality as a core breeding objective.

Although the observed compositional variation in our field-grown oat samples reflects contributions from both genotype and non-genetic factors (including year-to-year differences, growth system and management), the following section focuses on describing the overall range and magnitude of variability present across all cultivars and harvest years. This approach provides a comprehensive perspective on the diversity available for key nutritional traits within cultivated oat.

In the present study, there was almost a two-fold difference between the protein content of the oat variety at the lowest end of the range (11.5% Kwant, F21) and that at the highest end (22.9%, Caffreys No.1, F24). Based on independent studies, protein contents reported in the literature range from 10%^19,39^ to 24%^19^ on a dry basis, which is in agreement with our results. Reported ranges within single studies are often more restricted, mainly because there are often fewer cultivars analysed per study. Another important aspect pertains to the different conversion factors used in each study^21,37,39–42^. Several factors are being used, ranging from 5.34 to 6.25, due to the lack of a universal factor for the conversion from nitrogen content to protein in the case of oats. In the present study, a conversion factor of 5.83 was applied to nitrogen content^43^. Studies using higher or lower factors might tend to over- or under-estimate, respectively, the protein content. As an example, in the study of Nemeth et al.^20^, the reported protein range of eight cultivars can shift from 14.6–16.3% up to 17.1–19.1% by changing the conversion factor from 5.34 to 6.25. This illustrates how differences can be significant enough to result in non-overlapping ranges between studies. Similarly, changing the multiplication factor used from 5.83 to 6.25 would shift the observed protein content range from 11.5–22.9% to 12.6–25.1%, respectively. For the protein profile, the literature suggests that varieties with similar protein contents exhibit similar amino acid profiles. In a previous study, three Canadian naked oat varieties (AC Hill, AC Lotta, and AC Percy) with similar protein content were found to also have similar amino acid composition^44^, but another study^27^ observed significant variation in the protein patterns of 15 lines with varying protein content, which could result in a different amino acid composition.

The ash content in the samples varied within a narrow range (1.4–2.9% on a dry basis). This is mainly in accordance with other studies where the analysis of 5 to 18 cultivars resulted in an ash content range between 1.7 and 2.5% on dry basis^18,21^. The ash fraction is composed mainly of minerals such as calcium, magnesium, phosphorus and iron^45^. Despite the narrow variation ranges, the ash content can more than double between samples at each end of the range, which will result in a considerable increase in the micro-amounts of available minerals.

Fat content showed the greatest variability among the measured components (2.5–10% on dry basis; CV = 17.2–25.7%). While this level of variability is in accordance with earlier research on 50 cultivars grown in Sweden^46^, the ranges observed here remained below some of the highest values reported in the literature^47–50^. For example, certain oat varieties have been found to contain up to 15.5% fat (dry weight basis)^51^, and even 18.1% (wet weight)^52^. Among all components, fat content deviated the most from previously reported values. This notably lower range could be partly attributable to recent breeding efforts favouring lower oil content in oat cultivars, driven by technological requirements and the increasing use of oats in human consumption. Furthermore, Arendt et al.^53^ highlighted that reported high variation in literature stems from inconsistent methods and grain forms^53^. For instance, lipid content is lower when analysing husked grains, as oat husks contain few lipids. Since oats are also a grain of interest for animal feed, extensive research focused on the husked grain, as it would be consumed. When the focus shifted to human consumption, the compositional analysis was done more frequently on dehulled grains. Furthermore, methods that are based on distinct principles, such as solvent extraction, near-infrared spectroscopy or nuclear magnetic resonance spectroscopy (used here), are likely to result in different reported fat contents due to factors such as sensitivity, calibration, and measurement targets (i.e. direct fat extraction vs. indirect spectral or NMR signals). Previously, Zhou et al.^48^ mentioned that the Soxhlet method, often employed in fat determinations, can underestimate the total fat content due to incomplete extraction of polar and bound lipids^48^. In the present study, using the same method to evaluate all 95 samples, the upper range remained below that reported in the literature, and most varieties had average-to-low fat content compared to Husky, the reference variety in this study. While a low fat content is favoured from a processing point of view, higher contents may be more nutritionally valuable since oat is rich in unsaturated fatty acids^49^. Nevertheless, free fatty acids are susceptible to oxidation and consequent development of rancidity; therefore, high-oil varieties are only favoured where the oats are intended for animal feed^54^. Apart from the variation in total fat, fatty acid composition also varies^48^, with linoleic acid and oleic acid being the most abundant fatty acids in oats^49^.

Starch content as part of the oat grain is often overlooked in the literature. Research has typically been focused on the extracted or isolated starch and its properties^55–57^, rather than on starch as an integral component of the whole grain. In oats, starch is the most abundant and the least variable constituent^46^, accounting for approximately half the weight of the grains. This was reflected in our results, where the average starch content was 58.4 ± 3.6% (on a dry basis), and the variability was the lowest among the nutritional components (CV = 3.6–7.4%).

The fibre-rich fraction of the samples was calculated by difference from the rest of the components, resulting in a fraction that includes other material such as non-starch polysaccharides, resistant starch fractions, as well as other minor carbohydrates. This fraction showed a broad range (8.5–26.9%, on a dry basis), which is in accordance with the literature, when compared to the fibre content. Sterna et al.^58^ reported values between 13.7% and 30.2% (on a wet basis)^58^, but values as low as 2.1% (on a dry basis) have also been reported^39^. Variability in fibre content across studies can, in part, be attributed to the different methods used^59^; fibre can be determined by enzymatic-chromatographic techniques or calculated by difference (as fibre-rich fraction). Given the different nature of these methods and the sometimes-inconsistent definition of ‘fibre,’ reported results can vary substantially. Moreover, the sample sizes studied, which are often relatively small with fewer than 20 cultivars, may limit the representativeness of the results. β-glucan, the most distinctive fibre component in oats, has been reported to make up to 8%^22^ of oat groats' dry weight. In the present study, it ranged from 2.8 to 6.6% on a dry basis. The fibre and ash content can also be affected by the dehulling efficiency or potential damage. Higher residual husk contamination might elevate ash content in some cultivars, while more sensitive cultivars risk outer layer damage affecting fibre fractions and β-glucan. The dehulling time (2–3 min) was adjusted among cultivars to achieve complete hull removal while minimising groat damage. In contrast to stone and impact dehullers, which rely on abrasion and mechanical impact to separate hulls from kernels, the LPS1 uses high-pressure air to detach hulls with limited abrasion and breakage.

These results highlight the substantial nutritional diversity present in oat cultivars, providing a valuable resource for improving compositional traits to meet a range of needs from breeding programmes to human health and food product development, underscoring oat’s versatility and potential to better tailor variety selection for diverse applications.

Significant correlations among the main components analysed in the field-grown samples included in the present study were observed primarily for starch, which exhibited varying degrees of inverse relationships with fat and protein in most or all sample groups. The correlation matrices can be found in Fig. 1b and Table S4. This pattern is consistent with the compositional nature of cereal grains, where the major constituents compete for space and resources within the kernel. Indeed, Doehlert et al.^18^ mentioned that the negative correlation coefficients between starch and the other components can be explained by the starch being the major component of the grain^18^. Additionally, they noted that, since fibre is essentially carbohydrate, their negative relationship is expected, and this was also observed in other studies^18,60^. However, a smaller number of studies have reported no evidence of inverse relationships. For example, Lapveteläinen et al.^37^ found no relationship between starch and fibre (*r* = −0.11) and only a weak negative correlation between starch and protein (*r* = −0.30) when examining rolled oats from eight different cultivars^37^. Interestingly, separate investigations of 10 spring oat cultivars have even reported positive correlations between starch and protein^61^.

The variability in reported relationships highlights the complex interplay among oat grain constituents, which extends beyond starch to other key components such as protein, fat, and fibre. Earlier research has found a negative correlation between protein and fibre^34^, which was not observed in our results. Previously, Hartunian Sowa et al.^51^ and Peterson et al.^52^ reported an inverse relationship between lipids and starch when studying three and 25 oat lines with varying fat contents, respectively. Their results, however, are contradictory on the relationship between β-glucan and lipids, with the former finding an inverse relationship and the latter a positive one. A positive β-glucan-lipid relationship is also reported in earlier research on 50 samples grown in Sweden^46^ whereas other research reports no relationship at all (*r* = 0.11)^17^. About the relationship between protein and fat, literature findings are also inconsistent. Some research supports that fat content is positively correlated with protein^34,41,52^, but Lapveteläinen et al. and Ardayfio et al. found no correlation (*r* = −0.12, *r* = 0.19), which agrees with our results^17,37^. Lastly, a positive correlation between ash and protein has been documented in the literature^20,37^, and was also observed in our samples (*r* = 0.32 to 0.47).

Achieving a deeper understanding of the potential correlations among oat macronutrients can facilitate more targeted breeding and support strategies that promote the selection of oat varieties balancing agronomic performance, yield and nutritional quality.

This study clearly demonstrates a wide range of nutritional variability among oat cultivars, highlighting the considerable scope for improvement of the nutritional value and health-promoting properties of oats available to consumers. Husky has been cultivated in Ireland since 2007 and is recognised for its consistent performance in field trials both as autumn-sown and spring-sown oat. It is recurrently recommended by DAFM^13^ and is currently one of the only three recommended oat varieties in Ireland. Therefore, Husky was selected as a compositional reference cultivar in this study. Our analyses revealed that Husky ranked average for most nutritional components, with the exception of relatively higher starch and lower calculated fibre-rich fraction compared to the mean values across all samples analysed (Fig. 2). Although the calculated residual fraction cannot be equated with analytically defined total dietary fibre, higher values are likely to reflect greater amounts of fibre-related material in these oat samples. Considering that higher levels of β-glucan, protein and fibre are often favoured, these findings suggest that, from a strictly nutritional perspective, Husky may be less advantageous than other varieties included in this study. Examples of cultivars from the same sample group as Husky, which outperformed this cultivar in key health-related components, include Dyfed and Lipoplus, which exhibited higher β-glucan (6.6 and 5.3%, respectively, vs. 4.7%) and protein contents (19.4 and 19.1%, respectively, vs. 15.8%) when compared to Husky.

There was almost a two-fold difference between the protein contents of the varieties at the extremes of the range. This large variation translates to oat providing between 4.1 and 8.2 g of protein per typical serving (40 g oats, ~10% moisture content). To put this into perspective, a serving of oat at the lower end of this range contains roughly the same amount of protein as a slice of whole wheat bread (32.1 g of bread, 3.95 g protein), while at the higher end, it exceeds the protein content found in a serving of beans (90 g canned kidney beans, 6.58 g protein) (https://fdc.nal.usda.gov/). These findings highlight the potential of certain oat varieties to serve as a significant source of dietary protein, comparable to or even surpassing other commonly consumed plant-based foods. Beyond quantity, the quality and health-promoting potential of oat proteins are also noteworthy. Sanchez-Velazquez et al.^62^ reported that during digestion, oats release bioactive peptides of low molecular weight and free amino acids^62^. Therefore, it can be hypothesised that oat varieties with different protein patterns may produce distinct peptide profiles during digestion, potentially leading to variation in antioxidant activity and other functional properties. Further research into the peptide profiles of diverse oat cultivars could provide valuable insights into optimising both their nutritional and functional quality.

Although the variation of starch content was the lowest among the measured components (CV = 3.6–7.4%), the observed range (47.2–65.3%, on a dry basis) is considerable and may have physiological implications, particularly for energy density, glycaemic load, and postprandial glycaemic response. Moreover, other parameters, such as the composition and/or configuration of the starch granules (e.g. size, amylose content)^51^ as well as the interactions with other components native to the oat kernels (e.g. fibre^63^, β-glucan^24,64^, protein^64^, lipids^64^) may vary and influence the digestion rate of starch and consequently postprandial blood glucose concentrations.

High fibre is favourable from a nutritional perspective, and international authorities recommend a target intake value of at least 25 or 30 g/day, depending on the guidelines of each country^65^. In general, an adequate fibre intake is linked to a reduced risk of type-2-diabetes, obesity, cardiovascular disease, colorectal cancer, and normal bowel function^65^. Oat fibre, in particular, is considered to improve bowel function^3^. A proportion of the residual calculated fraction reported in the present study is expected to consist of fibre and fibre‑related fractions that may contribute to such effects; however, because this fraction was determined by difference rather than by an AOAC dietary fibre method, the specific physiological implications should be interpreted with caution.

High β-glucan content is specially desirable because of its health-promoting characteristics^6^. This soluble fibre can promote an increase in the viscosity of the chyme within the gastrointestinal tract, thereby delaying glucose absorption and ultimately attenuating postprandial glycaemic responses^2,66^. Moreover, a daily intake of 3 g of β-glucan has been shown to reduce blood cholesterol, which may, in turn, reduce the risk of coronary heart disease^1,67^, possibly via the same viscosity-increasing and matrix-entrapping mechanisms^68,69^. In practical terms, to obtain 3 g of oat β-glucan from a low β-glucan cultivar (2.5%), three portions of porridge (each made from 40 g of oat) would be required daily. On the contrary, just over a bowl porridge of a high β-glucan cultivar (50 g of oat, 6.0% β-glucan), would be enough for the same benefit. A porridge made from an average β-glucan content, such as the reference variety in this study, would lie somewhere in the middle, suggesting that most consumers, who typically consume just one bowl of porridge for breakfast, are unlikely to reach the recommended daily intake of 3 g of β-glucan if using average or low β-glucan oat varieties. This clearly demonstrates how the choice of oat varieties plays a crucial role in determining whether consumers can realistically achieve the recommended daily intake of β-glucan through typical dietary habits. Higher β-glucan varieties would make it more likely for consumers to meet these daily requirements and thus benefit from the associated health claims.

Finally, while the absolute ash content in our study varied within a narrow range (1.4–2.9%, on a dry basis), this level of variability can still have meaningful implications for consumers. Even minor differences in ash content may reflect substantial variations in the amounts of essential minerals available because these are typically required in microgram to milligram quantities per day in the human diet.

While the compositional reference sample, Husky, showed average compositional traits, several varieties exceeded it in key health-related components like β-glucan and protein, which resulted in substantial differences in the estimated nutritional value of a typical porridge serving. Variability in key components, such as protein and β-glucan, further highlights the complexity of oat composition and potential health implications. Importantly, cultivar choice can determine consumers’ ability to meet recommended intakes of beneficial components such as β-glucan, further highlighting the need to consider compositional diversity alongside agronomic traits to maximise oat’s nutritional and health potential. This can also be observed in Table 3, where the coverage of recommended dietary allowances is presented for Husky, Dyfed, and Lipoplus, alongside some commonly consumed foods. An oat serving (40 g) made from the cultivars Dyfed or Lipoplus includes more protein than one egg, and 64–80% of the recommended β-glucan amount necessary to lower the blood cholesterol. This further underlines the effect of variation of oat on the extent of the expected health benefits. While the agronomic performance of the cultivars was out of the scope of this study, preliminary data are promising. For instance, high single-year yield values can be seen in Table S1 for some cultivars such as Krezus (8.9 t/ha) and 14579Cn1/1/ (10.1 t/ha). Looking at the nutritional and agronomical data side-by-side reveals potentially competitive cultivars that can combine favourable nutritional composition with important agronomical traits, such as similar single-year yield to Husky (F22, 8.92 t/ha). For example, cultivar 14519Cn3/2 (sample-group F22, yield 8.03 t/ha) had a high protein content of 18.3 ± 0.1% on a dry matter basis. Similarly, Stork (F22, 8.8 t/ha) and 070-SO2011Au5 (F22, 10.26 t/ha) had high β-glucan contents of 5.8 ± 0.8 and 5.8 ± 0.4%, respectively, which is beneficial from a health perspective. Lastly, low fat content such as that of the cultivar 021-SO2011Au16 (F22, 8.7 t/ha, fat = 2.5 ± 0.4%) is preferred for processing.

**Table 3:** Daily values (DV) of main nutritional components and their percentage coverage by a typical serving of oats, white bread, eggs and black beans (wet weight/as-is)

Component	DV^a^	Husky	Dyfed	Lipoplus	Bread^b^ 1 slice (27.3 g)	Egg^b^ 1 unit, (50.3 g)	Black beans (canned)^b^ 1 serving (130 g)
		oat groats, 1 serving (40 g)					
		Nutrients covered by a portion of each food (% of DV)					
Protein	50 g	11.3	14.0	13.9	5.1	12.5	18.0
Total carbohydrates^c^ (incl. fibre)	275 g	9.9	9.6	9.2	4.9	0.2	9.3
Dietary fibre^d^	28 g						
- residual fraction by difference:		20.8	23.1	28.6			
- analytically defined:					2.2	<2.7	31.1
Fat	78 g	2.4	2.3	4.1	1.3	6.4	2.1
β-glucan	3 g	55.7	79.9	63.9	–	–	–

DVs are reference amounts of nutrients to consume daily, for adults and children older than 4 years, based on a 2000-kcal diet. The %DV indicates the contribution of a single serving to the total daily requirement.

^a^ DVs were obtained from the FDA, and the β-glucan recommended intake from EFSA.

^b^ Nutritional composition data from the USDA.

^c^ Total carbohydrates include sugars, starch, resistant starch, and dietary fibre and are compared with the DV of 275 g/day.

^d^ Dietary fibre values for comparator foods correspond to analytically defined dietary fibre and are compared with the DV of 28 g/day. For oats, the ‘residual fraction by difference’ is calculated as 100 minus the sum of analytically measured components and does not represent AOAC-defined total dietary fibre.

Taken together, our findings show that the nutritional diversity of oats, and consequently the potential health benefits for consumers, are intrinsically linked to agricultural selection pressures. This study quantifies compositional variability across 95 field-grown Irish oat cultivars, alongside paired field (2024) and glasshouse (2023) samples from 21 cultivars, providing comprehensive insight into compositional variability within this crop. Additionally, this study presents, for the first time, a direct comparison between glasshouse- and field-grown oat samples of 21 cultivars, offering valuable information on the influence of growth system, year and management on oat composition. The findings demonstrate substantial diversity in key nutritional components across oat varieties, in various growth systems and years. Most nutrient averages and ranges align well with values reported in the literature. Among the nutrients quantified, fat showed the largest variability, while starch content was the most consistent across varieties. These inter-varietal differences may have meaningful implications for consumer health. Accordingly, future studies should investigate the digestive process and potential health outcomes associated with consuming oat varieties with distinct compositions, particularly in relation to factors such as glycaemic response, gut microbiome modulation, and/or long-term health impacts, to provide a comprehensive understanding of the health implications of oat variety selection. Finally, given the direct influence of agricultural selection on nutritional diversity and consumer health, our study reveals a large scope for nutritional improvement. Overall, the broad ranges and overlapping values observed across different sample groups confirm the strong potential for selection and nutritional improvement in diverse systems and genetic pools. Strategic breeding initiatives can leverage this diversity to enhance the nutritional profile of oat, thereby supporting adherence to dietary recommendations for key health-supporting nutrients, such as protein and β-glucan, and ultimately contributing to a broader range of health benefits for consumers and food industry applications.

## Methods

### Oat samples

Ninety-five spring-type oat (*Avena sativa* L.) cultivars were included in this study. Seeds were grown across three distinct growing seasons and sites in Ireland as spring oats. The complete list of cultivars and their yield for the respective harvesting year is presented in Table S1. To control for inter-annual environmental variation, the field-grown samples were divided into three groups based on harvest year-site: the first group contained 33 cultivars grown in the field in 2021 (F21); the second group comprised 41 cultivars grown in the field in 2022 (F22); the third group consisted of 21 cultivars grown in the field in 2024 (F24). In addition to F24, glasshouse-grown samples of the same 21 cultivars were obtained in 2023 and included in the study; these are referred to as group G23.

Field trial in 2021 (F21) was conducted in Geradice, Co. Meath, Republic of Ireland (53 °27’N, 6 °43’W) on Grey Ashbourne soil. The 2022 (F22) and 2024 (F24) trial sites were located at Teagasc Oak Park Research Centre, Co. Carlow, Republic of Ireland (52.86 °N, 6.90 °W), with well-drained grey-brown podzolic soils of a clay-loam texture. Standard crop husbandry practices were followed for all field-grown samples as outlined by the Spring Oats Reference Guide (https://teagasccropreport.ie/reports/spring-oats-reference-guide). Briefly, nitrogen, phosphorus and potassium were applied based on land-use history and soil nutrient index level^70^. Crop was sprayed with a plant growth regulator and a fungicide to control lodging and powdery mildew, respectively. Plots (in 2021 and 2022) were harvested at maturity (grain moisture <20%) with a plot combine, providing harvested grain weight and a sample for each plot. Immediately after the harvesting, the moisture percentage was measured from a plot sample using the NIR (Near Infrared) grain moisture analyser. Any grain yield data presented in this manuscript were adjusted to 15% moisture (i.e. 85% dry matter) (Table S1). In 2024, oat genotypes were grown in rows and at maturity, they were harvested by hand, followed by hand threshing. Samples for analysis were air-dried and stored in cold storage, as necessary. In the glasshouse, cultivars were grown in pots of 20 cm diameter by 15 cm deep, filled with Jon Inns No.2 compost topped with adequate slow-releasing fertiliser (Osmocote). The growing conditions were maintained at 15–18 °C and a 16/8 h day and night cycle, supplemented by artificial LED light (Heliospectra AB).

This experimental design allowed for three main types of analyses: (1) an overview of the diversity found across all 95 field-grown cultivars; (2) comparison of cultivars grown within the same year (thereby minimising year-to-year effects); and (3) for a subset of 21 cultivars, comparison between glasshouse- (G23) and field-grown (F24) samples to provide a broader perspective on the potential performance of each genotype under different growth systems, year, and management. The variety Husky, included in group F22, was used as a reference variety as being one of the most commonly cultivated oat varieties in Ireland and recommended by the Department of Agriculture, Food, and the Marine, Ireland^13^.

### Preparation of samples

Samples were dehulled using a pneumatic laboratory testing huller LPS1 (Streckel & Schrader GmbH & Co. KG, Germany) operated at a constant air pressure of 100 PSI (7 bar). Dehulling time (2–3 min) was adjusted among cultivars to achieve complete hull removal while minimising groat damage. Following dehulling, any remaining empty hulls and unhulled grains were removed using a fan extractor and by manual inspection, respectively, so that only dehulled groats were retained for subsequent analyses. The cleaned dehulled samples (groats) were milled to pass through a 0.5 mm screen using a Retsch ZM200 ultra centrifugal mill (Retsch GmbH, Germany). The resulting oat flours were vacuum-packed in plastic film and stored at 4 °C until analysis.

### Compositional analysis

The compositional analysis aimed to quantify the major nutritional components of oat, specifically total moisture, ash, protein, fat, starch, β-glucan, and the calculated fibre-rich fraction. All compositional analyses were performed in triplicate (technical replicates) on flour milled from a single bulked sample of raw oat groats per cultivar as described below and results were reported as mean ± SD on a dry weight basis, unless stated otherwise.

Moisture and ash content were determined gravimetrically using a LECO TGA701 Thermogravimetric Analyser (Leco Instruments UK Ltd., Cheshire, UK). For moisture content determination, samples of 0.8–1 g were heated to 102 °C until constant weight. Subsequently, the samples were heated to 550 °C until constant weight to determine the ash content following the AOAC (Association of Official Analytical Chemists) official method 923.03^71^. Moisture content was used to calculate all other compositional parameters on a dry weight basis.

Protein concentration was determined using a LECO FP628 nitrogen analyser (LECO Corp., Michigan, USA) based on the Dumas method, following the AOAC method 992.15^71^. A nitrogen-to-protein conversion factor of 5.83 was applied^43^.

Total starch content was determined using a commercial kit (K-TSTA assay kit, Neogen® Megazyme International Ireland Ltd., Wicklow, Ireland) based on AOAC method 996.11^72^ and American Association of Cereal Chemists (AACC) method 76-13.01^73^. The manufacturer’s procedure for samples containing resistant starch (RTS-NaOH procedure) was followed, with minor modifications. Briefly, resistant starch in the sample (100 ± 3 mg) was pre-dissolved with 0.2 mL 80% aqueous ethanol and 2 mL cold 1.7 M NaOH, followed by starch hydrolysis by α-amylase and amyloglucosidase. The standard enzyme-incubation time was increased from 30 to 45 min to compensate for the lower heat conductivity of the plastic tubes used in place of glass. The assay was scaled down for the use of microplates instead of cuvettes by reducing the volumes for the subsequent reagent incubation and absorbance reading steps by ten-fold: duplicate 10 μL volumes from the sample tube and 10 μL volume from the sample blank were transferred to a 96-well microplate and incubated with 300 μL of glucose oxidase/peroxidase (GOPOD) reagent. The resulting D-glucose was quantified colourimetrically at 510 nm based on a glucose standard curve made for each microplate. Total starch content was then calculated by converting the determined glucose amount to starch using a factor of 0.9 to account for the molecular weight difference between glucose and its anhydrous form in starch.

Fat content was determined with a CEM ORACLE rapid NMR fat analyser paired with a CEM SMART 6 moisture analyser (CEM Corporation, North Carolina, USA). Samples (2–3 g) were first dried using the SMART 6, which employs dual-frequency energy (microwave and infrared) for moisture removal. Following standard sample preparation procedures, samples were immediately transferred to the ORACLE, which uses NMR technology to determine the fat content by isolating the proton signal of fat molecules from that of other sources. The specific instrument parameters were configured according to the manufacturer’s method note for “Oat Groats”^74^.

β-glucan content was determined using a commercial kit (K-BGLU assay kit, Neogen® Megazyme International Ireland Ltd., Wicklow, Ireland) following AOAC method 995.16^75^ and AACC method 32-23.01^73^ with minor modifications. Briefly, samples (100 ± 4 mg) were hydrated with sodium acetate buffer by boiling in water bath for 4 min (instead of the standard 3 min, to compensate for the lower heat conductivity of the plastic tubes used). The samples were then sequentially hydrolysed, first with lichenase to generate oligosaccharides, and after centrifugation to discard the insoluble material, by β-glucosidase to generate glucose. Incubation time after the addition of lichenase remained unchanged because the tubes had already equilibrated at the required temperature (50 °C) in a previous step. To adapt the assay to a microplate format, the volumes used for the incubation with β-glucosidase were reduced tenfold (10 µL each of supernatant and β-glucosidase were used instead of 100 µL). The same plate was then incubated with 300 μL of glucose oxidase/peroxidase (GOPOD) reagent. The resulting D-glucose was quantified colourimetrically as described above for the total starch procedure. The β-glucan content was then estimated by using a 0.9 conversion factor.

The residual fibre-rich fraction was calculated by difference, subtracting the sum of protein, fat, total starch, ash, and moisture from the total sample weight (100%). This fraction does not correspond to analytically defined total dietary fibre and likely comprises non-starch polysaccharides, resistant starch not quantified by the starch assay, other minor carbohydrate fractions, and cumulative analytical error.

### Statistical analysis

To provide a comprehensive overview of the dataset, descriptive statistics (mean, standard deviation (SD), coefficient of variation (CV)) were calculated using Excel 2016 (Microsoft Corp., USA) within and across groups. The CV for each nutritional component was calculated within each group as $({\rm{SD}}/{\rm{mean}})\times 100$ to express variability relative to the corresponding group mean. Pearson correlation coefficients were calculated in Excel and R (version 4.4.2, R Core Team, Vienna, Austria) within RStudio (version 2023.12.1, RStudio Team, Boston, MA, USA). Data visualisation was performed using R within RStudio and Excel.

All other statistical analyses were conducted using IBM SPSS Statistics software (version 29.0.1.0, IBM Corp., New York, USA), at a 5% significance level (*α* = 0.05), unless stated otherwise. Distributions of nutritional components for each field-grown sample group were visually inspected, and normality was further assessed using the Shapiro–Wilk test.

MANOVA/ANOVA was applied to technical triplicates (*n* = 3 per cultivar, dependent variables: protein, starch, fat, ash, β-glucan, calculated fibre-rich fraction) to assess analytical variation and rank cultivars descriptively; lack of biological replication (*n* = 1 bulked sample per cultivar) precludes formal inference on genotype differences. Partial eta-squared (*η*²_p, Wilks’ Lambda) quantifies effect magnitude. Univariate ANOVAs were performed where MANOVA results indicated significant differences to identify variables contributing to the overall effect, followed by Tukey post hoc tests. Assumptions of normality and homogeneity of variance of the triplicate observations were verified using the Shapiro–Wilk test and Levene’s test of equality of error variances, respectively. The normality assumption was satisfied in >90% of cases. Homogeneity of variances was not met, but MANOVA is considered robust to such violations. Box’s test of equality of covariance matrices was not computed due to the small sample size (*n* = 3). Such violations are common with small sample sizes (*n* = 3), and therefore, the analysis proceeded with caution. To control for confounding factors such as year-to-year variation, this statistical analysis was conducted separately within each sample group.

A paired *t*-test was used to compare differences in composition between cultivars grown under field and glasshouse conditions (F24 vs. G23), with normality of the differences confirmed by the Shapiro–Wilk test.

## Supplementary information

Supplementary material

## Data Availability

The data supporting the findings of this study, including all individual data points (presented as averages ± SD), are available within the Supplementary Materials of this article. Raw data are available from the corresponding author upon reasonable request.

## Code availability

The R code used for visualisation is available from the corresponding author upon reasonable request.
