# Supplementary material for: Comprehensive nutritional analysis of 95 oat cultivars reveals large variability in nutritional profile: protein, starch, fat, β-glucan and fibre
Source: NPJ Sci Food. 2026 Mar 17;10:145. doi: 10.1038/s41538-026-00800-z (PMC13144497; doi:10.1038/s41538-026-00800-z)
Supplement: Supplementary file 1 — Supplementary material [file 41538_2026_800_MOESM1_ESM.pdf]

Table S1. Oat cultivar names and their division into sample groups based on year of cultivation and growing conditions; namely, field 2021 (F21), field 2022 (F22), glasshouse 2023 (G23) and field 2024 (F24). The yield, as tonnes per hectare, is presented for the cultivation year of each sample group (single year).

Cultivars	Sample Group(s)	Yield (t/ha)	Cultivars	Sample Group(s)	Yield (t/ha)	Cultivars	Sample Group(s)	Yield (t/ha)	Cultivars	Sample Group(s)
AJ 130/163/25	F21	4.07	Pocspetri tf	F21	8.04	Banquo	F22	5.76	Avesta	G23 and F24
Anarcsi tf.	F21	6.07	Ravazdi tf	F21	6.47	Abundance	F22	5.32	Ayr ally	G23 and F24
Cacko	F21	4.49	Bogaty	F21	8.85	Bountiful	F22	5.91	Ayr line	G23 and F24
Dippawski	F21	6.67	Breton	F21	7.35	Dyfed	F22	6.01	Glasnevin ardi	G23 and F24
Dregelypalanki tf.	F21	4.94	Polar	F21	8.21	Tam finlay	F22	6.70	Stormont iris	G23 and F24
Ecsi tf.	F21	5.90	Ranch	F21	7.93	Sandy	F22	5.56	Ayr commander	G23 and F24
Gunhild	F21	5.72	Tenebra	F21	9.68	Tempo	F22	6.50	Caffreys No.2	G23 and F24
KENT BERLIE	F21	4.64	Dan	F21	8.36	SW Betania	F22	6.40	Glasnevin success No. 10	G23 and F24
Karcagi	F21	5.75	Conway	F22	8.28	Hedvig	F22	6.65	Victor	G23 and F24
Kwant	F21	7.53	Timpani	F22	9.36	Glasnevin Major	F22	6.27	Black rival NSG USA	G23 and F24
Lovaszpatonai sarga	F21	5.01	070-SO2011Au5	F22	10.26	Stormont Arrow	F22	5.33	Glasnevin triumph	G23 and F24
Nagyredei tf.	F21	5.60	021-SO2011Au16	F22	8.71	Stork	F22	8.84	Black spring	G23 and F24
Nidar Ii	F21	6.58	Milo	F22	6.17	Poretto	F22	5.43	Glasnevin success No. 3	G23 and F24
Zalalovoi tf.	F21	3.33	Blythe	F22	7.69	Lena	F22	6.31	Glasneving bonstar	G23 and F24
Irlande	F21	5.13	14579Cn1/1/1	F22	10.09	Jokikyla ME0501	F22	7.91	Caffreys No. 1	G23 and F24
Landsorte Jlitormo	F21	4.72	Caron	F22	8.77	Ayr Bounty	F22	5.69	Freddy TCFM002	G23 and F24
Stormogul II	F21	7.82	Maldwyn	F22	8.71	Miku	F22	8.51	TFCM006	G23 and F24
Strind	F21	6.82	Elen	F22	7.86	Cwal	F22	5.57	Brady	G23 and F24
Buillon	F21	9.11	Menai	F22	7.56	Ujszgedi	F22	7.55	Black oat	G23 and F24
Gagybatory K Tajfajta	F21	6.42	Milford	F22	6.64	Lipoplus	F22	4.38	Stormont grande	G23 and F24
Mersevati Tf	F21	4.17	14519Cn3/2	F22	8.03	Krezus	F22	8.90	Glasnevin potato	G23 and F24
Szegeti	F21	6.63	Scotch Berlie	F22	4.64	Husky	F22	8.92		
Furman	F21	7.63	Morlan	F22	3.14	Barra	F22	6.09		
Gniady	F21	4.68	Marvellous	F22	6.81	Isabel	F22	7.27		
Minue	F21	4.58	Melys	F22	8.44					

Table S2. Nutritional composition of field-grown oat samples. Results are presented as mean ± standard deviation of triplicate measurements on dry matter basis. Different superscript letters within in each column indicate statistically significant differences among cultivars within each field group (Tukey post-hoc tests, α = 5%).

(a) Field-grown cultivars harvested in 2021 (F21)

Cultivars	Protein (%)	Fat (%)	Ash (%)	Starch (%)	Fibre-rich fraction (%)	β-glucan (%)
AJ 130/163/25	15.46^{mn} ± 0.09	5.35^{f} ± 0.05	2.70^{fghi} ± 0.03	55.24^{abcde} ± 3.37	21.25^{abc} ± 3.44	2.80^{a} ± 0.34
Anarcsi tf.	13.35^{efg} ± 0.24	8.13^{kl} ± 0.07	2.68^{efghi} ± 0.09	53.08^{abc} ± 2.41	22.76^{abc} ± 2.60	3.69^{abc} ± 0.26
Cacko	13.07^{de} ± 0.03	8.07^{kl} ± 0.08	1.86^{a} ± 0.70	58.10^{bcde} ± 2.21	18.90^{abc} ± 2.33	4.37^{bcdef} ± 0.64
Dippawski	15.48^{mn} ± 0.29	5.39^{f} ± 0.05	2.61^{cdefghi} ± 0.03	59.86^{bcde} ± 3.28	16.66^{abc} ± 3.34	3.89^{abcd} ± 0.55
Dregelypalanki tf.	15.10^{lm} ± 0.10	8.99^{m} ± 0.06	2.82^{i} ± 0.11	56.61^{abcde} ± 3.49	16.48^{abc} ± 3.20	4.95^{cdef} ± 0.23
Ecsi tf.	14.64^{jkl} ± 0.09	8.58^{lm} ± 0.03	2.50^{bcdefghi} ± 0.03	61.79^{bcde} ± 1.02	12.49^{a} ± 1.03	3.69^{abc} ± 0.54
Gunhild	11.55^{a} ± 0.16	4.31^{ab} ± 0.19	2.24^{abcdefgh} ± 0.04	64.37^{de} ± 5.21	17.53^{abc} ± 5.22	5.08^{def} ± 0.76
Kent Berlie	13.51^{efgh} ± 0.18	7.11^{hi} ± 0.13	2.52^{bcdefghi} ± 0.27	51.06^{ab} ± 3.19	25.80^{bc} ± 3.05	3.79^{abcd} ± 0.53
Karcagi	11.57^{a} ± 0.09	8.63^{lm} ± 0.16	2.17^{abcdefg} ± 0.05	61.00^{bcde} ± 6.04	16.62^{abc} ± 5.95	3.70^{abc} ± 0.08
Kwant	11.48^{a} ± 0.15	4.14^{a} ± 0.14	2.36^{abcdefghi} ± 0.13	64.31^{de} ± 4.36	17.71^{abc} ± 4.59	4.15^{bcdef} ± 0.52
Lovaszpatonai sarga	14.70^{kl} ± 0.13	7.46^{ij} ± 0.10	2.67^{efghi} ± 0.04	55.97^{abcde} ± 3.20	19.20^{abc} ± 3.30	4.68^{cdef} ± 0.63
Nagyredei tf.	16.51^{o} ± 0.17	7.11^{hi} ± 0.02	2.75^{hi} ± 0.07	50.92^{ab} ± 2.05	22.71^{abc} ± 2.16	3.94^{abcd} ± 0.11
Nidar Ii	18.59^{q} ± 0.05	4.75^{bcd} ± 0.53	2.80^{hi} ± 0.07	55.06^{abcde} ± 5.29	18.81^{abc} ± 5.01	3.70^{abc} ± 0.22
Zalalovoi tf.	15.38^{m} ± 0.01	7.96^{jk} ± 0.11	2.69^{efghi} ± 0.01	54.08^{abcd} ± 1.77	19.89^{abc} ± 1.62	4.76^{cdef} ± 0.64
Irlande	15.94^{n} ± 0.15	6.70^{gh} ± 0.05	2.64d^{efghi} ± 0.03	56.96^{abcde} ± 5.21	17.76^{abc} ± 5.17	4.91^{cdef} ± 0.30
Landsorte Jlitormo	17.39^{p} ± 0.07	6.43^{g} ± 0.27	2.08^{abc} ± 0.04	47.17^{a} ± 4.41	26.93^{c} ± 4.62	3.32^{ab} ± 0.20
Stormogul II	13.71^{gh} ± 0.07	10.00^{n} ± 0.10	2.58^{cdefghi} ± 0.06	59.96^{bcde} ± 2.92	13.75^{a} ± 2.95	4.56^{bcdef} ± 0.49
Strind	16.76^{o} ± 0.06	4.45^{abc} ± 0.39	2.68^{efghi} ± 0.07	55.68^{abcde} ± 1.10	20.44^{abc} ± 1.49	4.91^{cdef} ± 0.39
Bullion	15.40^{m} ± 0.07	5.33^{ef} ± 0.28	2.29^{abcdefghi} ± 0.01	59.61^{bcde} ± 1.31	17.38^{abc} ± 1.17	4.46^{bcdef} ± 0.15
Gagybatory K Tajfajta	14.62^{jkl} ± 0.07	7.79^{jk} ± 0.09	2.72^{ghi} ± 0.07	59.45^{bcde} ± 5.67	15.42^{ab} ± 5.54	4.85^{cdef} ± 0.20
Mersevati tf.	13.51^{efgh} ± 0.10	6.19^{g} ± 0.20	2.64^{cdefghi} ± 0.11	55.44^{abcde} ± 3.64	22.22^{abc} ± 3.78	4.53^{bcdef} ± 0.29
Szegedi	14.88^{kl} ± 0.15	7.11^{hi} ± 0.08	2.52^{bcdefghi} ± 0.11	58.46^{bcde} ± 0.71	17.04^{abc} ± 0.46	4.01^{abcde} ± 0.19
Furman	12.53^{bc} ± 0.06	4.58^{abcd} ± 0.16	2.08^{abcd} ± 0.03	63.39^{cde} ± 3.56	17.43^{abc} ± 3.67	5.40^{f} ± 0.49
Gniady	12.34^{b} ± 0.17	4.78^{bcde} ± 0.05	2.34^{abcdefghi} ± 0.05	63.30^{cde} ± 0.47	17.24^{abc} ± 0.65	4.59^{bcdef} ± 0.26
Minue	15.56^{mn} ± 0.25	4.51^{abc} ± 0.13	2.50^{bcdefghi} ± 0.03	58.182^{bcde} ± 2.68	19.25^{abc} ± 2.96	5.31^{ef} ± 0.16
Pocspetri tf.	14.44^{jk} ± 0.12	7.04^{hi} ± 0.10	2.56^{cdefghi} ± 0.04	56.79^{abcde} ± 0.90	19.17^{abc} ± 0.68	4.76^{cdef} ± 0.37
Ravazdi tf.	14.45^{jk} ± 0.22	6.76^{gh} ± 0.13	2.37^{abcdefghi} ± 0.03	58.26^{bcde} ± 4.21	18.16^{abc} ± 3.99	4.42^{bcdef} ± 0.13
Bogaty	13.90^{hi} ± 0.19	4.93^{cdef} ± 0.14	2.65^{efghi} ± 0.07	59.03^{bcde} ± 3.21	19.49^{abc} ± 3.41	4.97^{cdef} ± 0.50
Breton	12.86^{cd} ± 0.37	4.03^{a} ± 0.07	2.29^{abcdefghi} ± 0.03	62.93^{cde} ± 3.65	17.89^{abc} ± 4.05	4.69^{cdef} ± 0.37
Polar	14.22^{ij} ± 0.10	6.57^{gh} ± 0.11	1.96^{ab} ± 0.54	62.14^{cde} ± 3.01	15.10^{ab} ± 3.08	4.85^{cdef} ± 0.68
Ranch	13.47^{efgh} ± 0.04	5.09^{def} ± 0.19	2.36^{abcdefghi} ± 0.10	63.76^{cde} ± 0.50	15.32^{ab} ± 0.42	4.79^{cdef} ± 0.03
Tenebra	13.18^{def} ± 0.08	4.97^{cdef} ± 0.14	2.14^{abcdef} ± 0.11	57.66^{abcde} ± 1.32	22.04^{abc} ± 1.17	4.93^{cdef} ± 0.13
Dan	13.65^{fgh} ± 0.10	5.33^{ef} ± 0.21	2.13^{abcde} ± 0.04	65.29^{e} ± 3.60	13.60^{a} ± 3.40	4.77^{cdef} ± 0.09

(Table S2 continued) (b) Field-grown cultivars harvested in 2022 (F22)

Cultivars	Protein (%)	Fat (%)	Ash (%)	Starch (%)	Fibre-rich fraction (%)	β-glucan (%)
Conway	14.54^{a} ± 0.05	6.86^{qr} ± 0.12	2.26^{bcde} ± 0.07	58.42^{bcd} ± 4.02	17.93^{abcde} ± 3.91	4.99^{abcdefg} ± 0.69
Timpani	14.40^{a} ± 0.10	5.99^{nop} ± 0.12	2.32^{bcde} ± 0.08	61.43^{cd} ± 1.04	15.85^{abcde} ± 1.03	4.50^{abcde} ± 0.33
070-SO2011Au5	14.93^{abc} ± 0.07	5.77^{klmnop} ± 0.28	2.32^{bcde} ± 0.08	59.85^{cd} ± 0.64	17.13^{abcde} ± 0.43	5.77^{defgh} ± 0.42
021-SO2011Au16	14.70^{ab} ± 0.08	2.45^{a} ± 0.41	2.30^{bcde} ± 0.04	60.58^{cd} ± 4.39	19.96^{abcde} ± 4.05	3.78^{a} ± 0.41
Milo	14.63^{a} ± 0.07	4.87^{cdefgh} ± 0.14	2.04^{abcd} ± 0.10	57.61^{abcd} ± 1.44	20.86^{abcde} ± 1.44	4.60^{abcdef} ± 0.20
Blythe	15.36^{cde} ± 0.18	5.40^{ghijklmn} ± 0.14	2.55^{bcde} ± 0.04	60.39^{cd} ± 3.81	16.30^{abcde} ± 3.76	6.12^{fgh} ± 0.78
14579Cn1/1/1	15.77^{efg} ± 0.07	7.57^{s} ± 0.30	2.39^{bcde} ± 0.04	49.48^{ab} ± 3.27	24.80^{e} ± 3.48	5.04^{abcdefg} ± 0.52
Caron	14.50^{a} ± 0.16	5.71^{jklmnop} ± 0.03	2.72^{cde} ± 0.01	59.03^{bcd} ± 3.53	18.04^{abcde} ± 3.67	5.44^{bcdefgh} ± 0.59
Maldwyn	16.49^{ij} ± 0.18	5.18^{fghijk} ± 0.28	2.16^{abcde} ± 0.07	55.08^{abcd} ± 1.53	21.09^{abcde} ± 1.34	4.08^{abc} ± 0.29
Elen	15.51^{def} ± 0.17	6.33^{pq} ± 0.32	1.94^{abc} ± 0.04	55.31^{abcd} ± 5.11	20.91^{abcde} ± 5.29	5.03^{abcdefg} ± 0.35
Menai	15.30^{cde} ± 0.05	4.38^{bc} ± 0.02	2.05^{abcd} ± 0.14	59.68^{cd} ± 1.43	18.58^{abcde} ± 1.43	4.47^{abcd} ± 0.32
Milford	17.36^{mn} ± 0.13	5.57^{ijklmno} ± 0.08	2.66^{cde} ± 0.12	58.35^{bcd} ± 3.99	16.07^{abcde} ± 3.97	5.67^{cdefgh} ± 0.83
14519Cn3/2	18.29^{pq} ± 0.05	6.16^{op} ± 0.20	1.94^{abc} ± 0.03	57.27^{abcd} ± 5.11	16.34^{abcde} ± 4.92	5.44^{bcdefgh} ± 0.29
Scotch Berlie	18.71^{qr} ± 0.12	5.71^{jklmnop} ± 0.21	2.60^{cde} ± 0.02	58.21^{bcd} ± 3.69	14.77^{abcd} ± 3.89	4.85^{abcdef} ± 0.35
Morlan	15.21^{bcd} ± 0.06	6.87^{qr} ± 0.24	2.37^{bcde} ± 0.07	55.53^{abcd} ± 0.82	20.02^{abcde} ± 1.04	6.10^{efgh} ± 0.78
Marvellous	16.44^{hij} ± 0.26	4.77^{cdefg} ± 0.03	2.41^{bcde} ± 0.08	57.81^{bcd} ± 0.30	18.57^{abcde} ± 0.27	4.96^{abcdefg} ± 0.52
Melys	14.71^{ab} ± 0.64	5.85^{lmnop} ± 0.13	2.28^{bcde} ± 0.19	60.53^{cd} ± 1.38	16.63^{abcde} ± 1.46	5.60^{cdefgh} ± 0.60
Banquo	15.31^{cde} ± 0.02	6.34^{pq} ± 0.14	2.27^{bcde} ± 0.15	57.91^{bcd} ± 2.96	18.17^{abcde} ± 3.03	5.88^{defgh} ± 0.37
Abundance	17.15^{klmn} ± 0.06	5.11^{defghij} ± 0.33	1.99^{abcd} ± 0.38	58.10^{bcd} ± 1.28	17.66^{abcde} ± 0.69	4.57^{abcdef} ± 0.19
Bountiful	18.08^{op} ± 0.14	7.03^{rs} ± 0.10	2.82^{de} ± 0.03	59.83^{cd} ± 1.75	12.24^{ab} ± 1.80	3.72^{a} ± 0.13
Dyfed	19.37^{s} ± 0.03	4.93^{cdefghi} ± 0.20	2.42^{bcde} ± 0.47	55.36^{abcd} ± 3.55	17.92^{abcde} ± 2.98	6.65^{h} ± 0.64
Tam finlay	16.25^{ghi} ± 0.14	5.14^{efghijk} ± 0.24	2.46^{bcde} ± 0.07	59.26^{cd} ± 1.91	16.89^{abcde} ± 1.79	4.45^{abcd} ± 0.30
Sandy	19.38^{s} ± 0.14	5.93^{mnop} ± 0.14	2.79^{de} ± 0.07	55.81^{abcd} ± 2.82	16.10^{abcde} ± 2.70	3.94^{ab} ± 0.34
Tempo	17.27^{lmn} ± 0.04	5.44^{hijklmn} ± 0.11	2.73^{cde} ± 0.02	55.48^{abcd} ± 1.91	19.08^{abcde} ± 1.85	6.45^{gh} ± 0.66
SW Betania	16.39^{hij} ± 0.03	5.59^{jklmno} ± 0.23	2.21^{bcde} ± 0.20	56.73^{abcd} ± 3.05	19.08^{abcde} ± 3.15	5.18^{abcdefgh} ± 0.52
Hedvig	15.82efg ± 0.06	4.48^{bcd} ± 0.17	2.35^{bcde} ± 0.26	59.3^{cd} ± 2.00	18.03^{abcde} ± 2.12	4.93^{abcdefg} ± 0.26
Glasnevin Major	16.74^{ijkl} ± 0.07	5.09^{defghij} ± 0.07	2.62^{cde} ± 0.02	52.13^{abc} ± 1.05	23.42^{de} ± 1.02	5.10^{abcdefgh} ± 0.21
Stormont Arrow	18.85^{rs} ± 0.16	4.10^{b} ± 0.26	2.94^{e} ± 0.03	62.66^{d} ± 3.23	11.46^{a} ± 3.30	4.43^{abcd} ± 0.20
Stork	15.22^{bcd} ± 0.15	5.74^{jklmnop} ± 0.15	2.41^{bcde} ± 0.14	56.51^{abcd} ± 3.35	20.13^{abcde} ± 3.33	5.80^{defgh} ± 0.78
Poretto	14.88^{abc} ± 0.15	4.72^{bcdef} ± 0.27	2.40^{bcde} ± 0.08	55.23^{abcd} ± 0.75	22.77^{cde} ± 0.53	4.91^{abcdefg} ± 0.37
Lena	17.59^{no} ± 0.08	4.52^{bcde} ± 0.18	2.70^{cde} ± 0.03	55.09^{abcd} ± 5.04	20.09^{abcde} ± 5.00	4.82^{abcdef} ± 0.53
Jokikyla ME0501	16.73^{ijk} ± 0.19	5.87^{lmnop} ± 0.10	2.50^{bcde} ± 0.05	61.27^{bcd} ± 4.99	13.62^{abc} ± 4.85	4.42^{abcd} ± 0.31
Ayr Bounty	17.30^{mn} ± 0.17	4.72^{bcdef} ± 0.23	2.23^{bcde} ± 0.62	52.68^{abc} ± 3.55	23.08^{cde} ± 4.24	5.54^{bcdefgh} ± 0.82
Miku	15.95^{fgh} ± 0.11	4.89^{cdefgh} ± 0.19	1.74^{ab} ± 0.52	55.50^{abcd} ± 1.38	21.92^{bcde} ± 1.57	4.69^{abcdef} ± 0.46
Cwal	14.41^{a} ± 0.24	4.48^{bcd} ± 0.07	1.36^{a} ± 0.56	60.68^{cd} ± 1.69	19.06^{abcde} ± 1.18	3.99^{bc} ± 0.50
Ujszgedi	16.87^{jklm} ± 0.14	6.02^{nop} ± 0.08	2.06^{abcd} ± 0.25	53.57^{abcd} ± 1.30	21.48^{bcde} ± 1.52	4.35^{abcd} ± 0.17
Lipoplus	19.14^{rs} ± 0.06	8.75^{t} ± 0.11	1.95^{abc} ± 0.93	48.07^{a} ± 3.18	22.10^{cde} ± 3.51	5.30^{abcdefgh} ± 0.73
Krezus	17.12^{klmn} ± 0.17	5.29^{fghijklm} ± 0.03	2.56^{bcde} ± 0.10	57.31^{abcd} ± 3.06	17.72^{abcde} ± 3.02	5.09^{abcdefgh} ± 0.36
Husky	15.81^{efg} ± 0.06	5.27^{fghijkl} ± 0.04	2.49^{bcde} ± 0.14	60.08^{cd} ± 3.00	16.35^{abcde} ± 4.06	4.68^{abcdef} ± 0.29
Barra	15.41^{cde} ± 0.16	4.41^{bc} ± 0.35	2.39^{bcde} ± 0.06	58.75^{bcd} ± 2.10	19.04^{abcde} ± 2.54	3.97^{bc} ± 0.30
Isabel	14.92^{abc} ± 0.15	4.76^{cdef} ± 0.12	2.41^{bcde} ± 0.07	59.18^{cd} ± 1.24	18.74^{abcde} ± 1.31	4.49^{abcd} ± 0.45

(Table S2 continued) **(c) Field-grown cultivars harvested in 2024 (F24)**

Cultivars	Protein (%)	Fat (%)	Ash (%)	Starch (%)	Fibre-rich fraction (%)	β-glucan (%)
Avesta	17.11^{de} ± 0.33	4.84^{bc} ± 0.21	2.37^{abc} ± 0.05	60.77^{abcde} ± 1.19	14.91^{bc} ± 1.73	6.26^{ab} ± 0.66
Ayr ally	18.69^{jkl} ± 0.11	5.82^{efg} ± 0.05	2.39^{abc} ± 0.33	58.24^{a} ± 0.72	14.87^{bc} ± 0.66	5.34^{ab} ± 0.74
Ayr line	17.41d^{efg} ± 0.20	6.07^{fghi} ± 0.05	2.07^{a} ± 0.08	59.72^{abcd} ± 1.00	14.73^{bc} ± 1.08	4.93^{ab} ± 0.63
Glasnevin ardi	19.16^{lm} ± 0.19	4.94^{bc} ± 0.11	2.31^{abc} ± 0.07	58.03^{a} ± 1.16	15.56^{c} ± 1.47	4.96^{ab} ± 1.85
Stormont iris	19.75^{m} ± 0.17	5.57^{def} ± 0.24	2.42^{abc} ± 0.28	57.47^{a} ± 1.10	14.78^{bc} ± 1.02	5.07^{ab} ± 0.43
Ayr commander	18.24^{hij} ± 0.19	4.03^{a} ± 0.07	2.39^{abc} ± 0.02	63.55^{bcde} ± 1.05	11.79^{abc} ± 0.75	4.73^{ab} ± 0.23
Caffreys No.2	18.46^{ijk} ± 0.04	6.82^{jk} ± 0.03	2.25^{abc} ± 0.10	57.01^{a} ± 1.65	15.46^{c} ± 1.74	6.19^{ab} ± 0.94
Glasnevin success No.10	17.65^{efgh} ± 0.09	5.90^{fgh} ± 0.07	2.36^{abc} ± 0.07	58.51^{a} ± 1.20	15.59^{c} ± 1.29	6.33^{ab} ± 0.92
Victor	18.86^{kl} ± 0.15	4.50^{ab} ± 0.03	2.28^{abc} ± 0.20	60.90^{abcde} ± 0.44	13.45^{bc} ± 0.42	4.92^{ab} ± 0.24
Black rival NSG USA	17.35^{def} ± 0.31	7.11^{k} ± 0.08	2.37^{abc} ± 0.10	59.44^{abc} ± 0.84	13.73^{bc} ± 0.44	5.75^{ab} ± 0.44
Glasnevin triumph	16.84^{d} ± 0.29	6.43^{hij} ± 0.51	2.34^{abc} ± 0.11	60.69^{abcd} ± 0.28	13.70^{bc} ± 0.53	6.02^{ab} ± 0.60
Black spring	17.57^{efg} ± 0.31	5.27cde ± 0.13	2.54^{bc} ± 0.08	59.31^{ab} ± 2.82	15.31^{c} ± 3.30	5.96^{ab} ± 0.80
Glasnevin success No. 3	18.28^{ijk} ± 0.09	5.12^{cd} ± 0.08	2.51^{bc} ± 0.08	60.33^{abcd} ± 0.78	13.76^{bc} ± 1.07	5.81^{ab} ± 0.16
Glasneving bonstar	17.91^{fghi} ± 0.18	4.07^{a} ± 0.02	2.42^{abc} ± 0.04	64.98^{e} ± 1.71	10.62^{ab} ± 1.74	4.82^{ab} ± 0.38
Caffreys No. 1	22.85^{n} ± 0.10	6.86^{jk} ± 0.39	2.63^{c} ± 0.06	59.19^{a} ± 2.79	8.48^{a} ± 2.47	4.50^{ab} ± 0.49
Freddy TCFM002	14.80^{b} ± 0.17	4.71^{bc} ± 0.27	2.27^{abc} ± 0.09	63.75d^{e} ± 1.16	14.46^{bc} ± 1.23	4.70^{ab} ± 1.11
TFCM006	13.87^{a} ± 0.33	4.82^{bc} ± 0.09	2.20^{ab} ± 0.04	63.70^{cde} ± 1.39	15.40^{c} ± 1.46	4.32^{a} ± 0.39
Brady	15.80^{c} ± 0.03	6.18^{ghi} ± 0.03	2.39^{abc} ± 0.02	61.19^{abcde} ± 0.85	14.43^{bc} ± 0.98	6.62^{b} ± 0.90
Black oat	15.80^{c} ± 0.18	6.61^{ijk} ± 0.08	2.36^{abc} ± 0.08	60.20^{abcd} ± 0.92	15.04^{bc} ± 0.85	6.58^{ab} ± 0.20
Stormont grande	17.99^{ghi} ± 0.12	6.96^{jk} ± 0.03	2.38^{abc} ± 0.08	58.79^{a} ± 1.66	13.89^{bc} ± 1.85	4.71^{ab} ± 0.33
Glasnevin potato	16.93^{d} ± 0.06	5.19^{cd} ± 0.23	2.54^{bc} ± 0.08	61.14^{abcde} ± 0.99	14.19^{bc} ± 1.05	5.37^{ab} ± 0.60

Table S3. Nutritional composition of glasshouse-grown oat samples. Results are presented as average content of triplicate measurements on dry matter basis ± standard deviation.

Glasshouse 2023 (G23)						
**Sample**	**Protein (%)**	**Fat (%)**	**Ash (%)**	**Starch (%)**	**Fibre-rich fraction (%)**	**beta-glucan (%)**
Avesta	21.23^{ij} ± 0.33	4.40^{ef} ± 0.14	3.44^{a} ± 0.24	53.26^{b} ± 3.47	17.67^{abcde} ± 3.91	4.23^{abcd} ± 0.77
Ayr ally	21.95^{jk} ± 0.09	4.11^{de} ± 0.25	2.92^{a} ± 0.11	49.23^{ab} ± 1.04	22.13^{abcdef} ± 0.79	3.78^{abcd} ± 0.11
Ayr line	22.34^{kl} ± 0.12	4.79^{f} ± 0.22	2.88^{a} ± 0.05	52.50^{b} ± 0.53	17.25^{abcd} ± 0.20	3.41^{a} ± 0.38
Glasnevin ardi	22.8^{5l} ± 0.12	4.20^{de} ± 0.10	2.47^{a} ± 1.44	55.12^{b} ± 3.56	16.31^{abc} ± 1.94	5.23^{d} ± 0.77
Stormont iris	22.40^{kl} ± 0.17	5.60^{g} ± 0.10	3.10^{a} ± 0.06	49.45^{ab} ± 1.96	18.61^{abcde} ± 0.75	4.71^{abcd} ± 0.27
Ayr commander	18.24^{bc} ± 0.03	3.87^{cd} ± 0.05	3.03^{a} ± 0.18	56.99^{b} ± 3.81	16.68^{abcd} ± 2.54	4.49^{abcd} ± 0.37
Caffreys No.2	19.66^{fg} ± 0.46	5.96^{gh} ± 0.12	2.73^{a} ± 0.15	59.29^{b} ± 7.54	13.90^{a} ± 7.10	3.87^{abcd} ± 0.78
Glasnevin success No. 10	20.58^{hi} ± 0.09	4.44^{ef} ± 0.14	2.99^{a} ± 0.10	49.01^{ab} ± 2.00	23.43^{bcdef} ± 1.74	3.68^{abcd} ± 0.30
Victor	20.96^{hi} ± 0.30	3.32^{ab} ± 0.02	2.82^{a} ± 0.04	48.64^{ab} ± 1.78	24.80^{cdef} ± 1.71	3.98^{abcd} ± 0.17
Black rival NSG USA	19.47^{efg} ± 0.05	4.88^{f} ± 0.05	2.43^{a} ± 0.74	48.89^{ab} ± 4.51	25.88^{ef} ± 2.88	4.58^{abcd} ± 0.64
Glasnevin triumph	20.18^{gh} ± 0.14	4.23^{de} ± 0.23	2.86^{a} ± 0.05	50.63^{ab} ± 3.19	21.83^{abcdef} ± 2.93	5.09^{bcd} ± 0.65
Black spring	19.19^{def} ± 0.15	3.78^{bcd} ± 0.25	2.77^{a} ± 0.04	49.31^{ab} ± 1.40	24.95^{def} ± 1.02	5.16^{cd} ± 0.48
Glasnevin success No. 3	21.36^{ij} ± 0.11	3.21^{a} ± 0.17	3.23^{a} ± 0.05	52.81^{b} ± 3.58	20.56^{abcdef} ± 1.94	3.73^{abcd} ± 0.50
Glasneving bonstar	20.89^{hi} ± 0.16	3.59^{abc} ± 0.20	2.98^{a} ± 0.06	50.72^{ab} ± 1.90	22.58^{bcdef} ± 0.75	3.78^{abcd} ± 0.13
Caffreys No. 1	22.69^{kl} ± 0.88	4.55^{ef} ± 0.03	2.99^{a} ± 0.03	41.02^{a} ± 2.30	28.21^{f} ± 1.14	3.55^{ab} ± 0.30
Freddy TCFM002	17.01^{a} ± 0.25	3.57^{abc} ± 0.18	2.62^{a} ± 0.08	57.55^{b} ± 4.39	18.30^{abcde} ± 3.24	3.62^{abc} ± 0.41
TFCM006	17.89^{b} ± 0.29	3.77^{bcd} ± 0.02	2.76^{a} ± 0.03	53.70^{b} ± 1.40	22.17^{abcdef} ± 1.47	3.95^{abcd} ± 0.20
Brady	18.36^{bcd} ± 0.06	7.00^{i} ± 0.01	2.81^{a} ± 0.11	54.19^{b} ± 3.34	18.29^{abcde} ± 2.90	4.73^{abcd} ± 0.74
Black oat	17.77^{ab} ± 0.26	6.15^{h} ± 0.05	3.00^{a} ± 0.02	59.65^{b} ± 8.33	15.96^{ab} ± 5.18	4.49^{abcd} ± 0.82
Stormont grande	18.37^{bcd} ± 0.02	5.69^{gh} ± 0.20	2.91^{a} ± 0.01	50.81^{ab} ± 0.81	22.48^{bcdef} ± 0.70	4.22^{abcd} ± 0.23
Glasnevin potato	18.77^{cde} ± 0.14	4.13^{de} ± 0.27	2.80^{a} ± 0.04	55.26^{b} ± 4.34	20.75^{abcdef} ± 1.89	4.14^{abcd} ± 0.54

Table S4. Correlation matrices (Pearson) of the nutritional composition of oat cultivars (%, dry matter basis). Positive values indicate positive correlations, negative values indicate negative correlations, and values near zero indicate little or no correlation. The asterisks indicate statistical significance (* $p < 0.05$; ** $p < 0.01$; *** $p < 0.001$).

(a) Group F21:

	Ash	*Fat*	*Starch*	*Protein*	*β-glucan*	*Fibre-rich fraction*
Ash	1.00					
Fat	0.20	1.00				
Starch	-0.41*	-0.32	1.00			
Protein	0.47**	0.04	-0.62***	1.00		
β-glucan	-0.14	-0.23	0.44**	-0.25	1.00	
Fibre-rich fraction	0.11	-0.12	-0.81***	0.26	-0.33	1.00

(b) Group F22:

	Ash	*Fat*	*Starch*	*Protein*	*β-glucan*	*Fibre-rich fraction*
Ash	1.00					
Fat	-0.03	1.00				
Starch	0.16	-0.45**	1.00			
Protein	0.32*	0.18	-0.33*	1.00		
β-glucan	0.12	0.28	-0.25	0.05	1.00	
Fibre-rich fraction	-0.43**	0.03	-0.77***	-0.26	0.13	1.00

(c) Group F24:

	Ash	*Fat*	*Starch*	*Protein*	*β-glucan*	*Fibre-rich fraction*
Ash	1.00					
Fat	0.03	1.00				
Starch	-0.08	-0.64**	1.00			
Protein	0.47*	0.17	-0.51*	1.00		
β-glucan	0.11	0.36	-0.33	-0.22	1.00	
Fibre-rich fraction	-0.48*	0.06	-0.34	-0.54*	0.43	1.00

(d) All field samples:

	Ash	*Fat*	*Starch*	*Protein*	*β-glucan*	*Fibre-rich fraction*
Ash	1.00					
Fat	0.13	1.00				
Starch	-0.09	- 0.34***	1.00			
Protein	0.19	-0.07	-0.27**	1.00		
β-glucan	-0.04	-0.05	0.09	0.22*	1.00	
Fibre-rich fraction	-0.16	0.01	-0.76***	-0.31**	-0.21*	1.00

(e) Group G23 - Glasshouse samples:

	Ash	*Fat*	*Starch*	*Protein*	*β-glucan*	*Fibre-rich fraction*
Ash	1.00					
Fat	-0.03	1.00				
Starch	-0.11	0.21	1.00			
Protein	0.24	-0.17	-0.55**	1.00		
β-glucan	-0.25	0.23	0.12	-0.11	1.00	
Fibre-rich fraction	-0.09	-0.37	-0.86***	0.15	-0.12	1.00

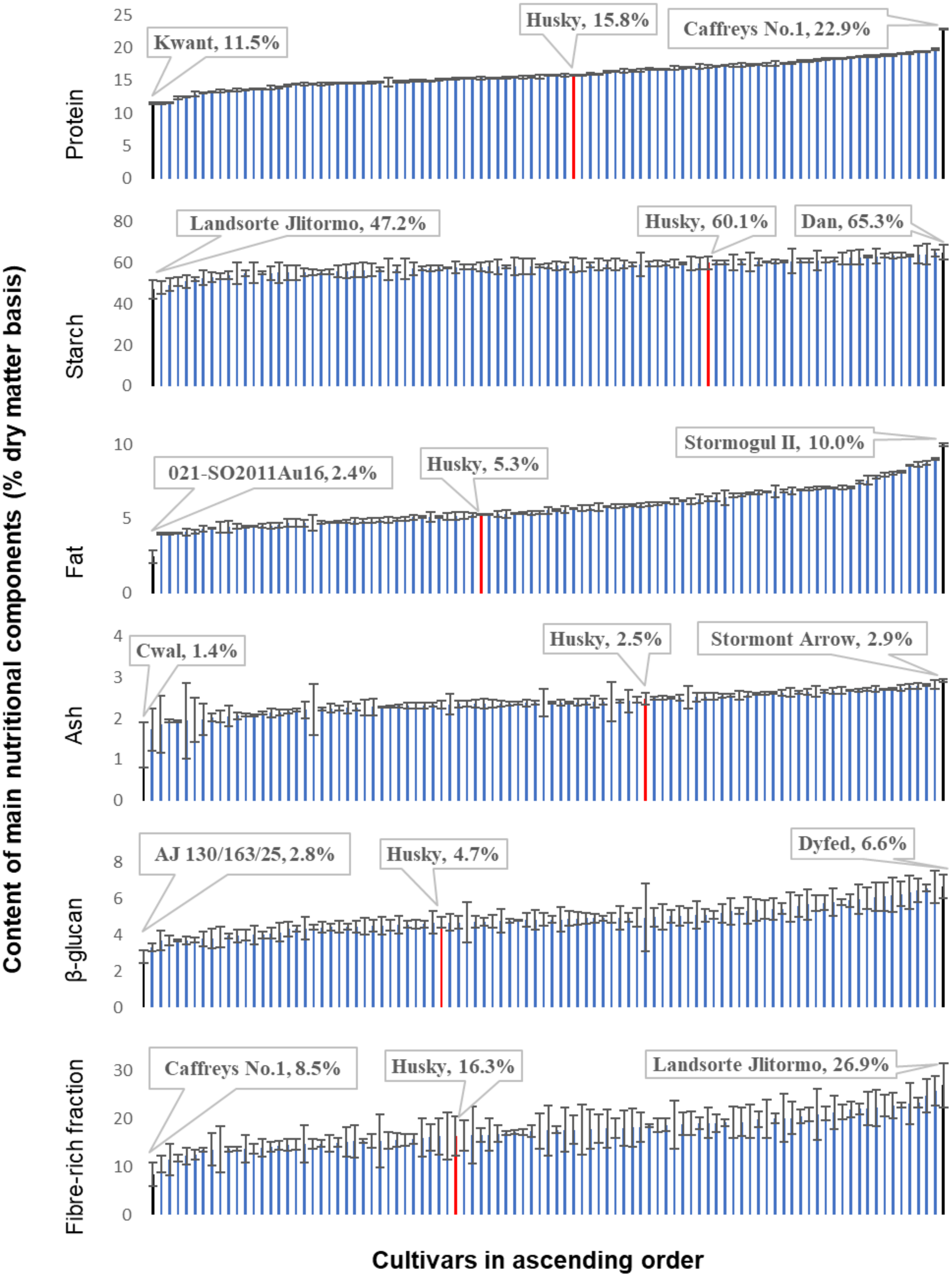

Figure S1. Macronutrient content of oat cultivars, expressed as percentage on dry basis with standard deviation (error bars). Cultivars arranged in ascending order. Highest, lowest, and reference (Husky) varieties are indicated in callouts; reference variety is shown in red.

Table S5. MANOVA and univariate ANOVA results for oat compositional traits.

MANOVA

Sample group	Statistic	Value	F	Hypothesis df	Error df	*p* value	η^2_p
**F21**	Wilks' Lamda	0.000	18.569	192.000	369.003	<0.001	0.903
**F22**	Wilks' Lamda	0.000	12.633	240.000	466.336	<0.001	0.864
**F24**	Wilks' Lamda	0.000	10.956	120.000	221.301	<0.001	0.845

ANOVA

Sample group	Dependent Variable	Type III Sum of Squares	df	Mean Square	F	*p* value	η^2_p
**F21**	Ash	6.383	32	0.199	6.662	<0.001	0.764
	Fat	252.564	32	7.893	261.127	<0.001	0.992
	Starch	1771.588	32	55.362	4.877	<0.001	0.703
	Protein	264.345	32	8.261	368.968	<0.001	0.994
	β-glucan	35.030	32	1.095	6.806	<0.001	0.767
	Fibre-rich fraction	1008.422	32	31.513	2.752	<0.001	0.572
**F22**	Ash	11.711	40	0.293	4.601	<0.001	0.692
	Fat	133.979	40	3.349	88.318	<0.001	0.977
	Starch	1182.186	40	29.555	3.490	<0.001	0.630
	Protein	263.946	40	6.599	253.390	<0.001	0.992
	β-glucan	62.630	40	1.566	6.658	<0.001	0.765
	Fibre-rich fraction	1006.194	40	25.155	2.889	<0.001	0.585
**F24**	Ash	.920	20	0.046	3.044	0.001	0.592
	Fat	55.560	20	2.778	82.573	<0.001	0.975
	Starch	285.603	20	14.280	7.579	<0.001	0.783
	Protein	205.973	20	10.299	263.435	<0.001	0.992
	β-glucan	31.736	20	1.587	3.002	0.001	0.588
	Fibre-rich fraction	187.133	20	9.357	4.396	<0.001	0.677
